# Characterization and Identification of Drought-Responsive ABA-Aldehyde Oxidase (AAO) Genes in Potato (*Solanum tuberosum* L.)

**DOI:** 10.3390/plants12223809

**Published:** 2023-11-09

**Authors:** Panfeng Yao, Chunli Zhang, Dan Zhang, Tianyuan Qin, Xiaofei Xie, Yuhui Liu, Zhen Liu, Jiangping Bai, Zhenzhen Bi, Junmei Cui, Jingwen Liang, Chao Sun

**Affiliations:** 1 State Key Laboratory of Aridland Crop Science, Gansu Agricultural University, Lanzhou 730070, China; yaopf@gsau.edu.cn (P.Y.); zhangchunl@st.gsau.edu.cn (C.Z.); zhangdan@gsau.edu.cn (D.Z.); qinty@st.gsau.edu.cn (T.Q.); xiexf@st.gsau.edu.cn (X.X.); lyhui@gsau.edu.cn (Y.L.); liuzhen@gsau.edu.cn (Z.L.); baijp@gsau.edu.cn (J.B.); bizz@gsau.edu.cn (Z.B.); cuijm@gsau.edu.cn (J.C.); 2 College of Agronomy, Gansu Agricultural University, Lanzhou 730070, China; 3 Planning and Finance Department, Gansu Agricultural University, Lanzhou 730070, China; liangjw@gsau.edu.cn

**Keywords:** potato, AAO family, drought stress, expression patterns

## Abstract

Abscisic acid (ABA) is an important stress hormone that affects plants’ tolerance to stress. Changes in the content of abscisic can have an impact on plant responses to abiotic stress. The abscisic acid aldehyde oxidase (AAO) plays a crucial role in the final step in the synthesis of abscisic acid; therefore, understanding the function of the *AAO* gene family is of great significance for insight into plants’ response to abiotic stresses. In this study, *Solanum tuberosum AAO* (*StAAO*) members were exhaustively explored using genome databases, and nine *StAAOs* were identified. Chromosomal location analysis indicated that *StAAO* genes mapped to 4 of the 14 potato chromosomes. Further analyses of gene structure and motif composition showed that members of the specific *StAAO* subfamily showed relatively conserved characteristics. Phylogenetic relationship analysis indicated that StAAOs proteins were divided into three major clades. Promoter analysis showed that most *StAAO* promoters contained cis-elements related to abiotic stress response and plant hormones. The results of tissue-specific expression analysis indicated that *StAAO4* was predominantly expressed in the roots. Analysis of transcriptome data revealed that *StAAO2/4/6* genes responded significantly to drought treatments. Moreover, further qRT-PCR analysis results indicated that *StAAO2/4/6* not only significantly responded to drought stress but also to various phytohormone (ABA, SA, and MeJA) and abiotic stresses (salt and low temperature), albeit with different expression patterns. In summary, our study provides comprehensive insights into the sequence characteristics, structural properties, evolutionary relationships, and expression patterns of the *StAAO* gene family. These findings lay the foundation for a deeper understanding of the *StAAO* gene family and offer a potential genetic resource for breeding drought-resistant potato varieties.

## 1. Introduction

During the growth of different crops, various abiotic stresses seriously impact their yield and quality. To mitigate these adverse environmental factors, plants have developed numerous adaptive mechanisms through the various signaling pathways that they rely on. Among all the signal pathways, hormone-regulated signal pathways play a more important role in plant resistance to adversity stress [1]. ABA, as an important secondary metabolite and stress hormone, plays crucial role in coping with adversity stress, promoting plant senescence, inhibiting plant growth, and regulating root development and fruit ripening [2]. The synthesis of ABA in plants is affected when plants are affected by drought, salt, low temperature, etc. [3]. In a stress environment, plants rapidly synthesize ABA, which can enhance the stress resistance of plants by reducing leaf swelling, inducing proline accumulation and soluble sugar accumulation, promoting active oxygen metabolism, reducing malondialdehyde accumulation, and regulating stomatal movement [4].

In recent years, studies on ABA have shown that there may be two biosynthetic pathways for ABA in higher plants [5]: (1) the direct pathway is the polymerization of three isopentane units to form the C15 precursor farnesyl pyrophosphate (FPP), which is formed directly from 15-carbon ABA by epoxidation and oxidation of FPP [6]; (2) in the indirect pathway, ABA is synthesized using carotenoids as precursors [7]. Zeaxanthin epoxidase (ZEP) catalyzes the formation of zeaxanthin from cyclic zeaxanthin, which is cleaved to flavin aldehyde by 9-cis-epoxycarotenoid dioxygenase (NCED). It is then converted into abscisic aldehyde by short-chain dehydrogenase (SDR), and finally oxidized by abscisic aldehyde oxidase (AAO) to ABA [6,8]. More and more evidence suggests that ABA may be synthesized through the C40 indirect biosynthesis pathway in higher plants, in which ZEP, NCED, SDR, and AAO are important enzymes in this pathway [9].

As a key gene in the last step of the ABA synthesis pathway, the biological function of *AAO* genes has been gradually studied in Arabidopisis [10], cotton [5], rice [8], maize [11], pea [12], and so on. *AtAAO4*, *A. thaliana* genes, played a critical role in delaying senescence in siliques by catalyzing aldehyde detoxification [13]. The heterologous expression of *AtAAO1* and *AtAAO3* not only produced hydrogen peroxide but also produced superoxide anions. Moreover, without AtAAO3 oxidation activity, *ataao3* mutant not only showed lower ABA, but also showed earlier senescence and dwarf symptoms [14]. *OsAAO3*, the *O. sativa* gene, was expressed in multiple tissues and organs (vascular, guard cell, germinated seed, root, leaf, and floral organ), and could be obviously induced by ABA and mannitol treatment [15]. The *osaao3* mutant exhibited decreased ABA levels, earlier seed germination, increased seedling growth, increased grain yield, and decreased drought tolerance compared to the wild-type [8]. Osmotic stress increased the transcriptional level and activity of TaAAO2/3 from *Triticum aestivum* leading to ABA biosynthesis, which brought about a faster closure of the stomata upon increasing osmotic stress [16]. The *AhAAO2* in *Arachis hypogaea* could be induced by exogenous ABA and overexpression of *AhAAO2* in Arabidopsis improved ABA levels and drought resistance [17]. In summary, the *AAOs* plays an important and complex role in the process of plant growth and adaptation to adversity.

Potato (*Solanum tuberosum* L.) is the fourth largest crop in the world and is indispensable for global food security. However, it is often affected by various stresses during its growth stage. For Gansu, a province with less precipitation in China, drought is one of the main abiotic stresses faced by potatoes in this area [18]. Therefore, our team is committed to the cultivation of drought-resistant potato varieties, drought-resistant gene discovery, and regulation mechanism analysis. To date, some progress has been made in studying the hormone regulation of potato development and stress response [19]. However, there is limited knowledge regarding the characterization of potato *AAO* genes and their response patterns to abiotic stresses. To address this gap, we performed genome-wide identification and molecular characterization of potato *AAO* genes. Meanwhile, the expression profiles of *StAAO* genes under different abiotic stress conditions were also analyzed. This work provides a potential genetic resource for drought resistance breeding.

## 2. Results

### 2.1. Identification of StAAO Genes

In this study, based on the conservation of AAO protein sequences in existing species, nine *AAO* genes were identified from the potato genome using the BLAST method and randomly named *StAAO1*-*StAAO9* (Table S1). Subsequently, the physical and chemical properties of StAAO proteins were predicted using the tools at the ExPasy website (http://web.expasy.org/protparam, accessed on 5 March 2023) and TargetP-2.0 (https://services.healthtech.dtu.dk/services/TargetP-2.0/, accessed on 7 March 2023), including coding sequence length (CDS), isoelectric point (pI), molecular weight (MW), and subcellular localization. The length of proteins sequence ranged from 470 (StAAO7) to 582 (StAAO4) amino acids (aa), with an average of 552 aa. Their molecular weights ranged from 52.94 kDa (StAAO7) to 66.22 kDa (StAAO4), with an average of 62.12 kDa. Their pI ranged from 7.72 (StAAO3) to 9.28 (StAAO5), with an average of 8.96. The results of subcellular localization prediction showed that four StAAO proteins (StAAO4/7/8/9) were located in cytoplasm, two StAAO3/6 were located in the Golgi apparatus, and the other three were located in the extracellular space (StAAO1), mitochondria (StAAO2), and endoplasmic reticulum (StAAO3) (Supplementary Table S1).

### 2.2. Structural Analysis and Motif Composition of StAAO Genes

**Figure 1:**
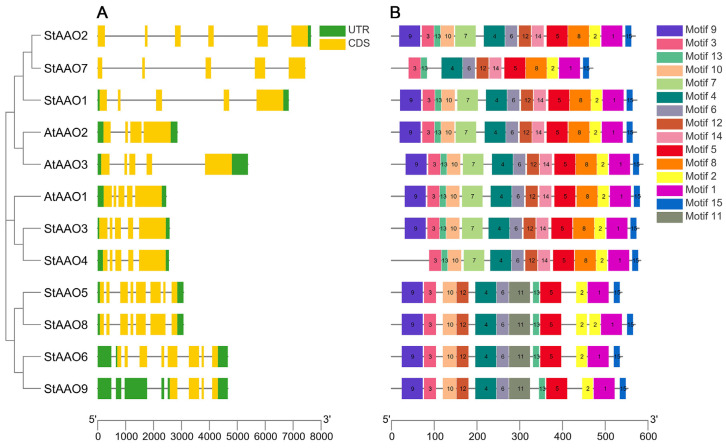
The gene structure and motifs of the StAAO family. (**A**) The green box, yellow box, and black line, respectively, represent untranslated 5′ and 3′ regions, exons, and introns. (**B**) Fifteen conserved motifs of StAAO proteins were listed.

To further understand the structural composition of the *StAAO* genes, the exon/intron structure of the *StAAOs* was constructed by comparing the genomic DNA sequences of the *StAAO* genes (Figure 1). The results demonstrated that the coding sequences of all *StAAO* genes contained introns, with the number of introns ranging from three to eight. These findings indicate significant structural differences among the nine *StAAO* genes (Figure 1A). In addition, we can see that *AAO* genes clustered in the same subgroup had more similar gene structures, such as *StAAO3/4* and *AtAAO1*, *StAAO5* and *StAAO8*. This suggests that *AAO* genes with similar structures may have some similarities in function. Interestingly, all *StAAOs* contain introns, which is quite different from the gene structure of *ZmAAO* [11]. This implies that there may be significant differences in the evolution of *AAO* between potatoes and maize.

To further explore the characteristic regions and predict the function of StAAO proteins, 15 conserved motifs were identified through MEME and further visualized by TBtools (Figure 1B). The number of motifs in StAAO proteins varied from 11 to 14, of which 6 motifs were functionally defined. In fact, the motif of StAAOs was extremely conserved, and motif 3/4/6/5/2/1/15 exists in all StAAO proteins. Except for StAAO7, all the other StAAOs contained at least 12 Motifs. Of course, there were also some variations between different subgroups. For example, Motif 7, 8, and 14 only existed in clade I and II, while motif 11 was unique to group III. In addition, we found that there were some structural differences even among the proteins clustered in the same subgroup. For example, StAAO8 in clade III contained two motif 2s, and StAAO4 in clade I and StAAO7 in clade II had no motif 9 compared with other members. Based on the above results, we speculated that these specific motifs may play an important role in the gene function of different subfamilies.

### 2.3. Phylogenetic Analysis Divided StAAOs into Three Sub-Groups

**Figure 2:**
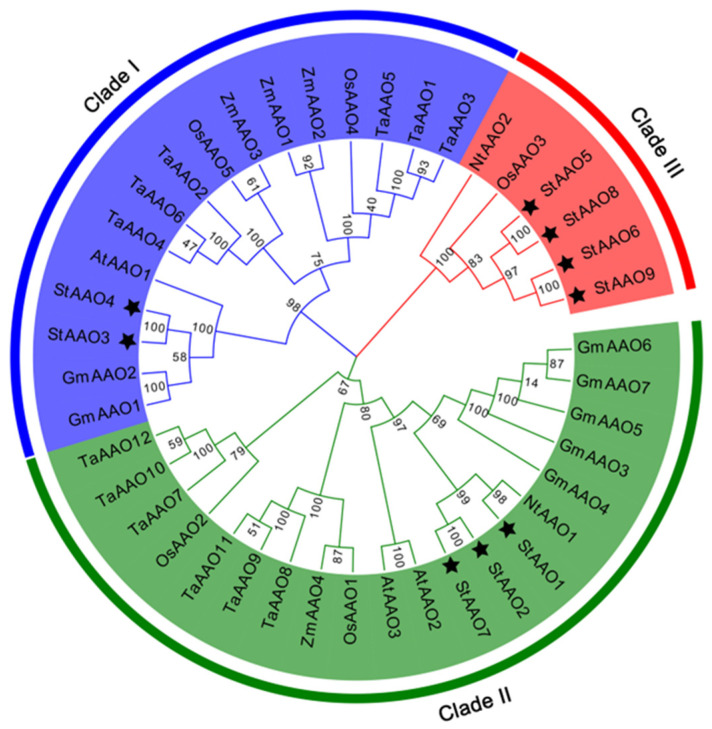
The evolution tree of Arabidopsis, rice, tobacco, wheat, maize, soybean, and potato were constructed by MEGA7.0. The maximum likelihood method was used to construct the phylogenetic tree, and the default parameter value was set to 1000. The black pentagram represents the potato AAO proteins.

In order to investigate the evolutionary relationship, a total of 42 AAO proteins from 7 different plant species were subjected to phylogenetic analysis, including 9 StAAOs, 3 AtAAOs, 5 OsAAOs, 2 NtAAOs, 7 GmAAOs, 4 ZmAAOs, and 12 TaAAOs (Figure 2). The tree showed that all AAO proteins were divided into three major clades (I, II, and III), which was consistent with previous publications. Two StAAOs (StAAO3 and StAAO4) were classified into clade I, three StAAOs (StAAO1, StAAO2, and StAAO7) were classified into clade II, and four StAAOs (StAAO5, StAAO6, StAAO8, and StAAO9) were classified into clade III. Most StAAO proteins were concentrated in clade III. However, it was observed that most AAO proteins were concentrated in clade I and clade II, and there were only six proteins in clade III, accounting for only 14.3% of the total AAO proteins. The results indicated that the evolution of AAO in potato might be quite different from other species. From the perspective of the overall branches, all AAOs in this tree do not show a clear clustering trend between monocotyledonous and dicotyledonous plants. All three clades contain both monocotyledonous and dicotyledonous plants, which was similar to previous reports [20]. In addition, there is a certain pattern within each sub-clade. For example, in clade I, the dicotyledonous plants Arabidopsis, potato, and soybean *AAO* genes cluster together in a small clade, while the monocotyledonous plants wheat, corn, and rice cluster together in another small cluster. Similar trends were also observed in clade II. It is worth noting that there were some differences in clade III.

### 2.4. Chromosomal Distribution, Gene Duplication, and Synteny Analysis of StAAO Genes

**Figure 3:**
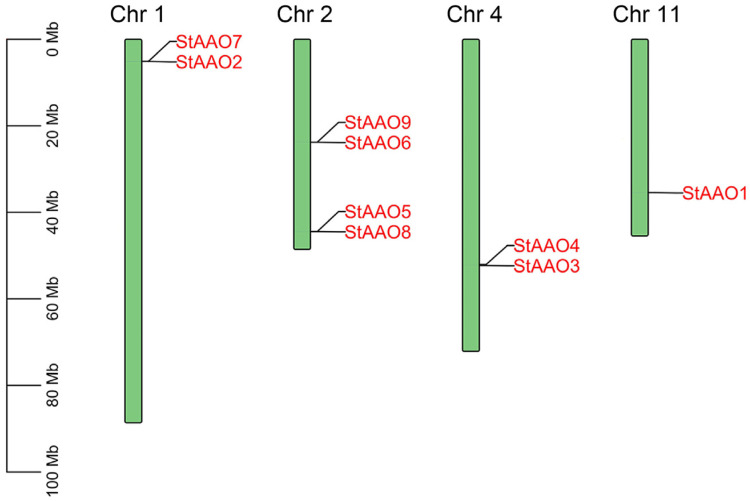
Schematic diagram of chromosome distribution of the *StAAO* gene. The chromosome number is displayed on the left side of each chromosome.

A chromosomal distribution map of *StAAO* genes (Figure 3) was drawn using the online potato genome database (http://spuddb.uga.edu/dm_v6_1_download.shtml, accessed on 18 April 2023). A total of 9 *StAAO* genes were unevenly distributed in 4 of 12 potato chromosomes. Among of them, Chr2 contained the most *StAAO* genes (four, ~44%), followed by Chr1 and Chr4 (three, ~22%), and Chr11 containing only one (~11%). Interestingly, the distribution of *StAAO* genes on the chromosome was mostly concentrated at both ends of chromosome, and only *StAAO6* and *StAAO9* were distributed in the middle of Chr2. In addition, the analysis results showed that there were three pairs of alleles in the StAAO gene family, namely *StAAO2*-*StAAO7*, *StAAO6*-*StAAO9*, and *StAAO5*-*StAAO8*.

**Figure 4:**
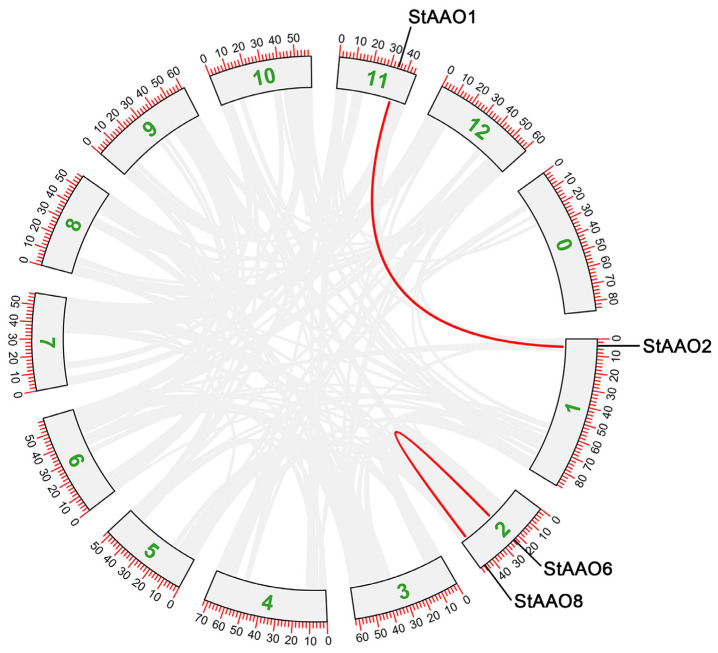
The relationship between chromosomes of StAAO chromosomes was visualized through multiple collinear scanning toolkits (MCScanX) and TBtools. Gray lines represent collinear blocks within the potato genome, while the red line highlights AAO gene pairs. Boxes represent different chromosomes of potatoes.

As gene replication events play an important role in the occurrence of new gene functions and the expansion of family, we further analyzed the replication of *StAAO* genes, including tandem and segmental duplication events (Figure 4). The results showed that two *StAAO* genes were clustered into two tandem repeat event regions (*StAAO6*/*StAAO8*) in Chr2, indicating that they were the hot spots of *StAAO* gene distribution. Meanwhile, a pairs of segmental duplication genes were detected between two chromosomes: Chr1 (*StAAO2*)/Chr11 (*StAAO1*). In short, it is possible that some *StAAO* genes arose through gene duplication and that these duplication events were the main drivers of *StAAO* evolution.

### 2.5. Evolutionary Analysis of StAAO Genes among Multiple Species

**Figure 5:**
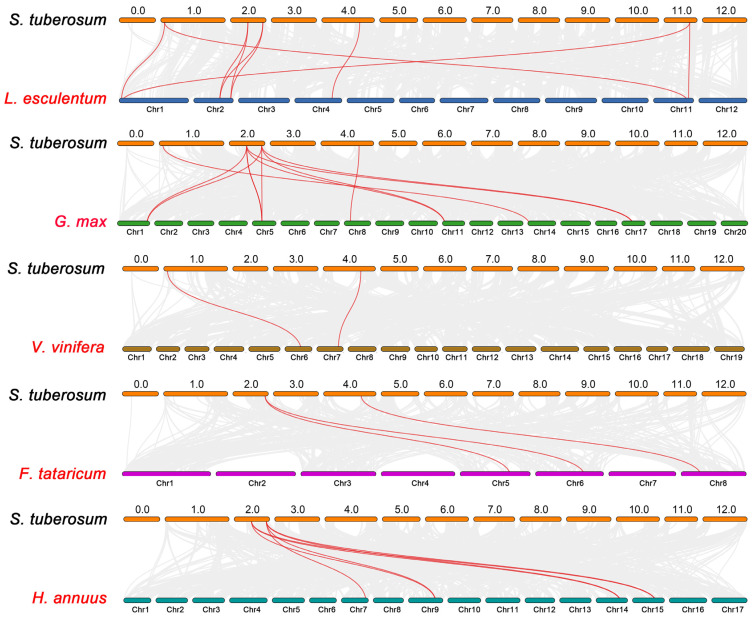
Collinearity analysis of *AAO* genes between potato and five other plants. Gray lines represent collinear blocks in the potato genome and other plant genomes, and red curves represent collinear *AAO* genes.

To further investigate the evolutionary relationship of the potato *AAO* gene family, a phylogenetic tree was constructed using five dicotyledonous species, namely tomato, soybean, grape, tartary buckwheat, and sunflower (Figure 5). A total of five *StAAO* genes were homologous to tomato, followed by four in soybean, and only two in grape, buckwheat, and sunflower. The number of AAO homologous pairs between potato and the five species (tomato, soybean, grape, tartary buckwheat, and sunflower) were 9, 11, 2, 3, and 7, respectively. Furthermore, we found that *StAAO8* has the most synthetic gene pairs (12) among all genes, followed by *StAAO6* (9), *StAAO2* (4), *StAAO4* (2), *StAAO1* (2), and *StAAO3* (1), suggesting that *StAAO8* may play a key role in the evolution of the *AAO* subfamily.

### 2.6. Analysis of Cis-Acting Elements of StAAO Genes

**Figure 6:**
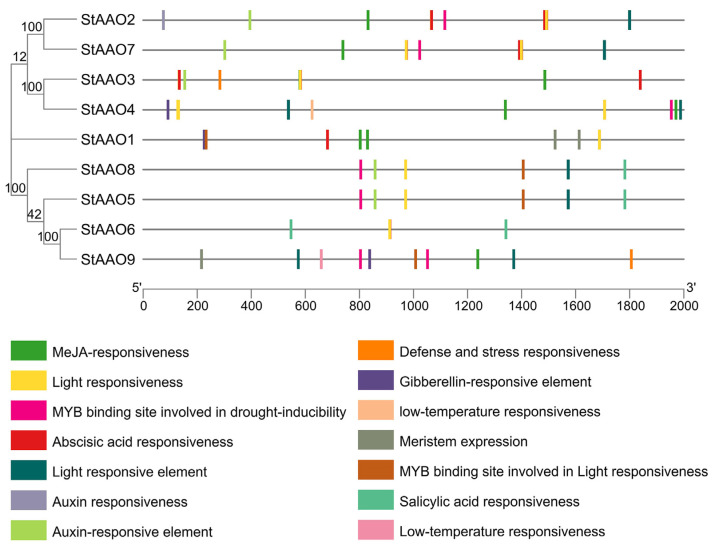
The element analysis of *StAAO* promoters. The environmental response elements are shown.

**Figure 7:**
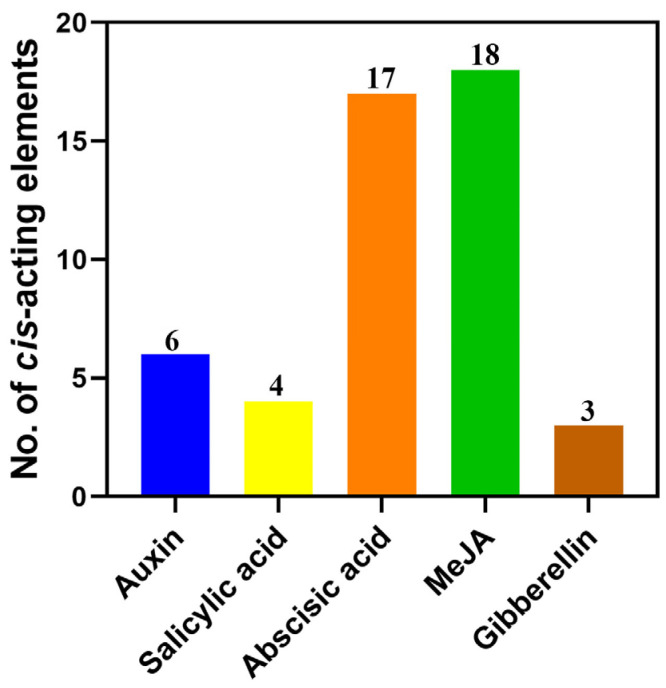
Number of hormone response elements in potato AAO family gene promoters.

*Cis*-acting elements play a crucial role in the transcription and expression of genes and can provide a variety of functions to regulate plant growth and its adaptation to the environment. To further reveal the characteristics of *StAAOs* and predict the possible regulatory pathways involved, the types and numbers of elements in *StAAO* promoter sequences were analyzed. We mainly focused on the environmental response elements (Figure 6). Overall, the *StAAO* promoters mainly included hormone-responsive elements (TGACG-motif, TCA-element, ABRE, etc.), stress-responsive elements (MBS, TC-rich repeats, LTR, etc.), metabolism-related-responsive elements (AACA_motif and MBSI), and a large number of light-responsive elements (MRE, G-box, TCT-motif, etc.). Of all the hormone response elements, the number of MeJA-responsive elements (CGTCA-motif) was the largest (18), followed by abscisic-acid-responsive elements (ABRE, 17), auxin- responsive elements (TGA-element, 6), salicylic acid (TCA-element, 4), and gibberellin-responsive elements (GARE-motif, 3) (Figure 7). The hormone response elements were found to be highly prevalent among the promoters of *StAAOs*. For example, ABA and MeJA response elements were present in almost all promoters. The promoters of *StAAO1* and *StAAO6* genes contained the most types of elements. The above results suggested that *StAAOs* may function through some certain hormone response pathway.

### 2.7. The Response of StAAO Genes to Drought Stress

**Figure 8:**
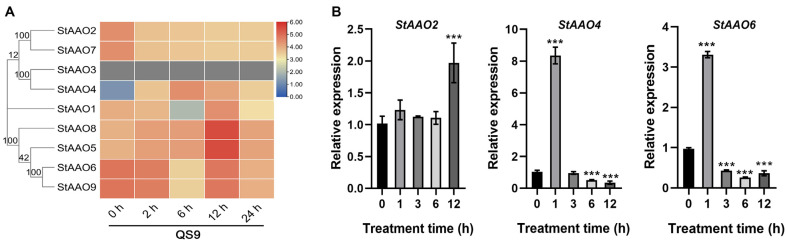
The response of *StAAO* genes to drought stress. (**A**) Heatmap showing transcriptome data of *StAAOs* after 0, 2, 6, 12, and 24 h of drought stress. Heatmap was generated based on log2 FPKM. (**B**) Expression levels of *StAAO2*, *StAAO4*, and *StAAO6* were analyzed by qPCR after 0, 1, 3, 6, and 12 h of drought stress. The 0 h was taken as a reference to determine relative mRNA level under stress conditions. Data represent the means ± SD of three replicates. Data points marked with asterisks (*** *p* ≤ 0.001) indicate a statistically significant difference between control and stress treatments.

Based on the analysis of the promoter sequence elements of *StAAO* genes, we speculated that *StAAO* genes may respond to various hormones and abiotic stresses. Due to our long-term focus on exploring drought resistance genes and studying their regulatory mechanisms in potato, we first analyzed the expression profile of *StAAO* genes under drought stress. Transcriptome analysis showed that there were some differences in the response of *StAAO* genes to drought stress (Figure 8A). Based on the response pattern to drought stress, *StAAO* genes can be divided into three distinct categories. The first type had a higher level of local expression but showed a downward trend with the extension of treatment time, mainly including *StAAO2/7/6/9*. The second type of genes showed an opposite trend with the extension of drought stress, mainly including *StAAO1/4/5/8*. It is worth noting that *StAAO4* had the lowest expression level in both varieties before drought treatment, but its response to drought stress was the most intense, and the maximum changes reached 16- and 30-fold during the treatment, respectively, which was much higher than other *StAAO* genes. However, the expression level of *StAAO3* was too low to detect.

Three *StAAO* genes that displayed substantial responsiveness to drought stress were subsequently validated using qRT-PCR, revealing two distinct response patterns (Figure 8B). Although the expression of the *StAAO2* gene fluctuated slightly 6 h before drought stress, there was no significant difference; a significant difference appeared after 12 h, reaching 1.97 times the pretreatment level. *StAAO4* and *StAAO6* showed a consistent expression trend under drought treatment, both of which were significantly up-regulated after 1 h of stress, reaching 8.21- and 3.32-fold higher than 0 h, respectively; after 3 h, it began to significantly decrease and almost reached the minimum value after 12 h.

### 2.8. Expression Patterns of Drought-Stress-Related StAAO Genes in Different Potato Tissues

**Figure 9:**
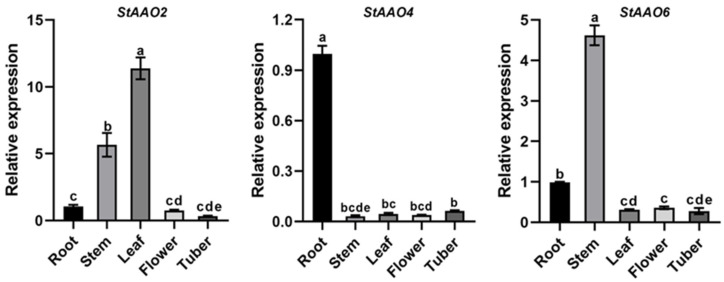
Expression analysis of *StAAOs* in five tissues of flowering potato. The expression level of all genes in roots was set to 1. Data represent the means ± SD of three replicates. The letters at the bar chart top indicated significant differences between different organizations (α = 0.05).

To further investigate the physiological functions of *StAAO* genes, the expression patterns of three drought-stress-related *StAAO* genes (*StAAO2/4/6*) in multiple potato tissues were analyzed (Figure 9). The three *StAAO* genes were expressed with different degrees in the five tested tissues, but the expression trends were different. The expression level of the *StAAO2* gene was highest in the leaves, followed by the stems, and was significantly lower in the roots, flowers, and tubers than in the leaves and stems, and there was no significant difference among the three tissues. *StAAO4* was highly expressed in the roots, which was significantly higher than that in the other four tissues, reaching 31.3, 21.9, 25.6, and 15.8 times the levels found in the leaves, stems, flowers, and tubers, respectively. The expression of *StAAO6* was the highest in the stems, followed by the roots, followed by the leaves, flowers, and tubers. The results indicated that the expression of *StAAO* genes was variable in different tissues.

### 2.9. Response of Drought-Stress-Related StAAO Genes to Various Abiotic Stresses

**Figure 10:**
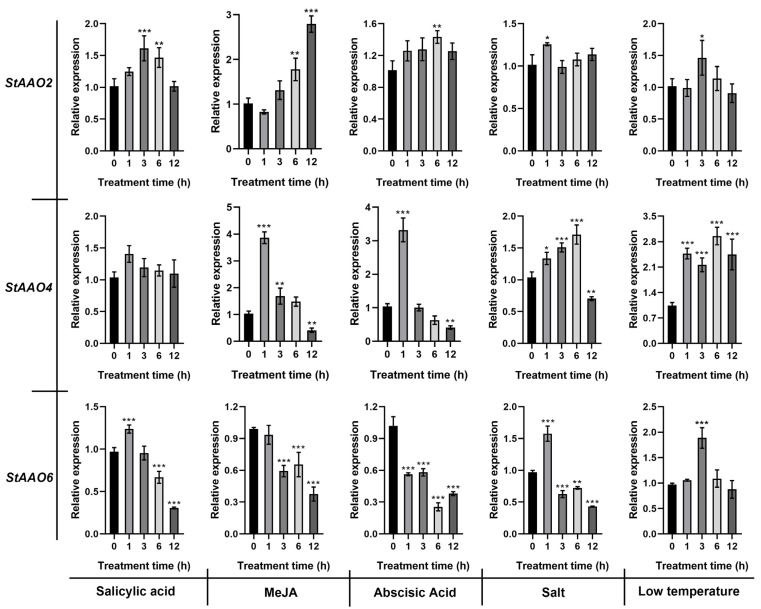
The relative expression level of *StAAOs* under low temperature, salt, SA, MeJA, and ABA treatments. The expression level of all genes at 0 h was set to 1. Data represent the means ± SD of the three replicates. Data points marked with an asterisk (* *p* ≤ 0.05, ** *p* ≤ 0.01, and *** *p* ≤ 0.001) indicate a statistically significant difference between the control and stress treatments.

The analysis of cis-acting elements showed that most *proStAAOs* contained abiotic stress and hormone response elements. Therefore, we further analyzed the response of three drought-responsive *StAAO* genes (*StAAO2/4/6*) to SA, MeJA, ABA, salt, and low temperature (Figure 10). *StAAO2* showed a relatively slow response to hormones, and its response to MeJA and ABA reached a significant level at 6 h. In addition, the response of *StAAO2* to salt and low temperatures was weak, and the differences were significant only after 1 h and 3 h of treatment, respectively, and then tended to revert to the expression level before treatment. *StAAO4* showed a more obvious response than *StAAO2*. Except for a non-significant difference in the response to SA, significant differences were observed in the other four stress conditions within 1 h. It is worth noting that the response of *StAAO4* to hormones and stresses exhibited an opposite trend. The response trends of *StAAO6* to the three hormones were basically consistent; fluctuating after 1 h of stress and being continuously down-regulated after 3 h. Under the salt condition, the expression level of *StAAO6* was significantly up-regulated to 1.6 times that of the control after 1 h, but then it significantly decreased and reached the minimum after 12 h. *StAAO6* showed significant differences to low temperature only at 3 h.

## 3. Discussion

Studies have shown that *AAO*, as a key gene in the last step of the ABA synthesis pathway, was usually induced by various stress conditions [10,21]. At present, the research on *AAO* genes has mainly focused on the functional study of a single gene in Arabidopsis, rice, maize, and so on [22]. With the improvement of potato whole genome sequencing data, the complete sequences of different types of genes can be easily retrieved from the genome. Systematic analysis of the molecular characteristics of potato *AAO* family genes and their response patterns to various hormones and abiotic stresses will help us to rapidly screen candidate genes that respond to abiotic stress in potato. AAO-mediated synthesis of ABA is an important part of the complex network between hormone signals and abiotic stress signals [23,24]. In our study, we identified a total of nine *StAAO* genes. The StAAO protein sequences have high similarity to the AAO family in Arabidopsis and rice. The data suggested that different AAO proteins might function in the same manner under different microenvironments. Genome-wide analysis showed that 5, 3, 7, 4, and 6 *AAO* genes were identified in the genomes of rice, Arabidopsis, soybean, corn, and sorghum, respectively [20]. The number of *AAO* genes in potatoes is higher than that in the aforementioned species. Compared to diploid plants such as Arabidopsis, maize, soybean, and sorghum, potatoes, as tetraploid plants, have a higher number of chromosomes. This may result in the *AAO* genes in potatoes having more copies during the evolutionary process. In addition, the AAO protein sequence exhibits high conservation, suggesting a closer evolutionary relationship among *AAO* genes in these species (Figure 1). Our phylogenetic tree did not show clear clustering patterns based on monocotyledons and dicotyledons for all AAOs as a whole. However, within each small clade, the AAO proteins of different species show a relatively obvious clustering pattern based on monocotyledons and dicotyledons. Based on this, we speculate that after the separation of monocotyledons and dicotyledons in evolution, the AAO proteins in three different clades have undergone significant differences in function, while the AAO proteins of different species within the same subclade gradually exhibit similar structures or similar functions during the long-term evolutionary process.

The expression or transcription of genes is initiated through the upstream regulatory promoter region, which can be considered a combination of many cis-acting regulatory elements fused with the minimal basic starting unit [25]. The various combinations of regulatory cores endow promoters with characteristics of strength, spatiotemporal specificity, and response to stimuli [26]. Therefore, analyzing the regulatory elements of the target gene promoter can better help us to predict its response to various stimuli. The analysis of *StAAO* gene promoters showed that there were many regulatory elements related to phytohormones, stress, and development (Figure 6). ABRE elements are mainly involved in plant responses to ABA signals [27,28]. Previous studies have shown that the presence or absence of ABRE elements may affect its inducibility for most *AAO* genes [29]. Most *AAO* gene promoters contain one or more ABRE, indicating that they may be responsive to ABA [30]. In this study, 88% (8/9) of *AAO* gene promoters contained at least one ABRE element, among which *StAAO2* and *StAAO7* had the highest number with four elements each. In addition to maintaining the dynamic balance of ABA, *AAO* genes are also widely involved in disease resistance as well as biotic and abiotic stress responses, which is consistent with the abundance of cis elements related to stress in their promoters. *StAAO* promoters contained many stress-related regulatory elements, including SA-responsive TCA-elements and SARE; MeJA-responsive elements, the TGACG-motif; defense- and stress-responsive elements, LTR and MBS; and the flavonoid biosynthesis element, MBSI (Figure 7). Among them, seven of nine *StAAO* gene promoters contained at least one stress response element, MBS, LTR, or TC-rich repeats. Therefore, according to the above results, we can speculate that *StAAO* genes may participate in potato stress resistance through the ABA hormone signaling pathway.

Besides ABA, published studies have shown that ethylene, GA, MeJA, and SA also affect the expression levels of several *AAO* genes across various plant species [22]. Moreover, *AAO* genes have also been implicated in various biotic and abiotic stress responses [31], indicating crosstalk between stress and hormone signaling [24]. Physiological effects of various phytohormones are known to be manifested, in part, by altering expression of genes responsive to these hormones. In order to study whether *StAAO* genes are also involved in crosstalk with other phytohormone signals as well to various environment stresses, their expression in response to various external stimuli was analyzed. Here, ABA treatment sharply effected the expression of *StAAO4* and *StAAO6*, slightly inducing up-regulation *StAAO2*. Interestingly, the expression pattern of *StAAO* genes under SA was similar to ABA treatment. SA and ABA are known to play key roles in plant defense, and SA- and ABA-dependent defense pathways exhibit crosstalk with each other [32], which suggested that *StAAO* might participate in the crosstalk between SA- and ABA-dependent defense pathways. *AhAAO2* was dominantly expressed in leaves, and its transcript level was greatly increased under exogenous ABA application; overexpression of *AhAAO2* in Arabidopsis led to improved ABA levels and drought tolerance after drought treatment [33]. The Arabidopsis *AAO3* knockout mutant *aao3* exhibited earlier senescence compared with the wild-type during normal growth or upon application of UV-C irradiation. Different aldehydes accumulated prominently in *aao3* mutants compared with WT leaves under normal growth conditions, upon UV-C irradiation and after exogenous aldehyde application [24]. In this study, *StAAO4* and *StAAO6* were markedly induced in response to various phytohormones and abiotic stress treatments, particularly *StAAO4* (Figure 8 and Figure 10). Studies have shown that most *AAO* genes that respond to hormone or adversity stress belong to clade III [34]. However, our results showed that the clade I and II genes of *StAAO* also exhibited a similar expression trend. The altered expression response to certain hormones may be due to the difference in plant species, suggesting novel functions in adaptation to changed circumstances in the process of evolution.

Determining the spatio-temporal expression patterns of candidate genes can partially predict their biological functions in tissue/organ development [35,36]. A large number of studies on the expression pattern of *AAO* genes in different plants provided rich references for analyzing *AAO* in potato. In Arabidopsis, *AAO3* is abundant in leaves and is necessary for drought-inducible ABA accumulation in the leaves, while other AAOs would be involved in ABA synthesis in the roots or seeds [37]. In the present study, the transcription levels of candidate *StAAOs* in five tissues were determined. Similar to other plant species, *StAAO* genes were differentially transcribed. All three tested *StAAOs* had low expression levels in tubers (Figure 9). *StAAO4* was specifically expressed in the roots, at a significantly higher level than in the other four tissues. For a long time research has indicated that the root tips were the main sites of ABA biosynthesis and the synthesized ABA is transported to the target tissues [38]. This result is similar to the *AtAAO3* that is involved in abscisic acid (ABA) biosynthesis in response to drought stress [38]. The above results indicated that *StAAO4* gene may participate in the response of potato to abiotic stress through ABA synthesis. Potato has indeterminate growth habits, and excessive vegetative growth will have a negative impact on potato production [39], so the potato canopy can usually be controlled by applying excessive plant growth regulation. In Arabidopsis, the overexpression of most *AAO* genes changed the balance of active auxin in vivo, forming a dwarf phenotype [14]. This gives us an important clue that the aerial part of potato can be controlled by regulating the expression of *StAAO*. Our data showed that *StAAO6* was mainly expressed in stems, which indicated that it might play a role in potato growth and development.

On the other hand, experiments with many plants showed that *AAO* not only participates in the regulation of plant growth and development but also plays an important role in plants’ response to abiotic stresses. Overexpression of the *AAO* gene in tobacco inhibited germination and seed yield upon salt stress [40]. In another report, up-regulation of cucumber *AAO* gene expression reduced the apoplastic AA redox state and oxidative stress tolerance in tobacco plants [41]. Transgenic tobacco overexpressing the *AAO* gene from cucumber displayed an increase in lower stomatal conductance and higher water content as compared to WT [42]. In contrast, suppression of the tomato *AAO* resulted in AA accumulation in fruits and enhanced drought stress tolerance by higher photosynthetic capacity [43]. The opposite roles of *AAO* genes in different species indicated that *AAO* has diverse functions across plant species. In this study, *StAAO4* was not only induced by drought stress but also significantly responded to low temperature, salt, ABA, and MeJA stress. Therefore, considering the specific expression pattern of *StAAO4* in roots, it is plausible to speculate that *StAAO4* may participate in regulating the potato’s response to abiotic stress through the promotion of ABA synthesis. Of course, the more accurate biological function and molecular mechanism still needs further experimental verification. Our study comprehensively analyzed the molecular characteristics and expression characteristics of the *AAO* family genes and selected *StAAO4* that significantly responded to drought stress, laying a very good theoretical foundation for further research on drought resistance mechanisms and drought resistance molecular breeding of potatoes.

## 4. Materials and Methods

### 4.1. Query and Identification of StAAO Genes

The potato genome sequence relevant files (fasta and GFF format) were obtained from Potato Genomics Resource (http://spuddb.uga.edu/, accessed on 10 January 2023), and four AtAAO protein sequences were acquired from TAIR (https://www.arabidopsis.org/, accessed on 13 January 2023). StAAO candidate proteins were obtained through whole genome sequence alignment by two BLASTP methods in TBtools [44]. The candidate genes were searched by BLASTP using a score value of ≥100 and e-value ≤ e^−10^ [45]. The candidate proteins were further identified by conserved domains (CD-Search, https://www.ncbi.nlm.nih.gov/Structure/bwrpsb/bwrpsb.cgi, accessed on 28 January 2023) and conserved motifs (MEME, http://meme-suite.org/tools/meme, accessed on 28 January 2023) [45]; then the final *StAAOs* members were determined and randomly named.

### 4.2. The Basic Bioinformatics Analysis of StAAOs

Molecular weight (MW) and isoelectric point (*p*I) were analyzed by EXPASY (http://web.expasy.org/protparam/, accessed on 13 March 2023). The annotation of intro/exon and chromosome distribution was analyzed and visualized by TBtools software (v1.09876). For cis-acting elements analysis, the 2000 bp sequences upstream of the start codon of *StAAOs* were considered as promoters and extracted by TBtools (v1.09876). The types and quantities of cis-acting elements were predicted by PlantCARE (http://bioinformatics.psb.ugent.be/webtools/plantcare/html/, accessed on 24 March 2023) and visualized by TBtools (v1.09876).

### 4.3. Phylogenetic Analysis of StAAO Proteins

For the phylogenetic tree, the AAO proteins of Arabidopsis [10], rice [46], tobacco [40], wheat [16], maize [11], soybean [47], and potato were used. The AAO phylogenetic tree of the above species was constructed by MEGA7 [48]. In MEGA7, the maximum likelihood method and Poisson model were selected, the option in Rates among Site was Gamma Distributed (G), the Site Coverage Cutoff parameter was set to 95%, and 1000 Bootstrap tests were carried out [45]. The resulting tree was initially generated in “Newick” format and subsequently refined using EvolView (https://evolgenius.info//evolview-v2/#login, accessed on 6 May 2023). All the sequences used to construct the phylogenetic tree are shown in Supplementary Table S3.

### 4.4. Intraspecific/Interspecific Collinearity Analysis of StAAO Genes

We utilized the Multiple Collinearity Scan Toolkit (MCScanX) to detect gene duplication events [49]. For intraspecific collinearity analysis, the potato genomic sequence and GFF annotation file (Genome assembly: SolTub_3.0) were self-aligned by TBtools (v1.09876). For interspecific collinearity analysis, the genomic sequence and GFF annotation files (tomato, soybean, grape, tartary buckwheat, and sunflower) were downloaded from EnsemblPlants (http://plants.ensembl.org/index.html, accessed on 17 May 2023) and compared with the potato genome sequence by TBtools (v1.09876). Then, the syntenic analysis maps of the Dual Systeny Plotter procedure (https://github.com/CJ-Chen/TBtools, accessed on 17 June 2023) were constructed to determine the syntenic relationship between *StAAO* genes and *AAO* genes in other selected plants [50]. Finally, the collinearity results of *StAAO* genes were visualized by TBtools (v1.09876).

### 4.5. Expression Analysis of StAAO Genes

Potato tissues (root, stem, leaf, flower, and tuber) at the flowering stage were collected and frozen rapidly in liquid nitrogen for expression analysis of *StAAO* genes. The expression data of *StAAO* genes under drought stress (heatmap) came from our previous transcriptome data [51]. For the data of the heatmap, a log2 transformation was applied to the FPKM data.

For treatments, the potato “Qingshu 9” (QS9, drought-tolerant) test-tube seedlings were cultured in an artificial climate room (16 h/8 h light/darkness, 25 °C, and 60% relative air humidity) for 30 days [52]. The seedlings were treated with liquid 1/2 MS medium with or without treatment. The final concentrations of hormones (ABA, MeJA, and SA), mannitol, and NaCl were 100 μM, 200 mM, and 100 mM, respectively. For low-temperature stress, potato test-tube seedlings were moved to a chamber at 4 °C. After 0, 1, 3, 6, and 12 h of treatment, the seedlings were collected, frozen rapidly in liquid nitrogen, and stored at −80 °C for RNA extraction according to manufacturer’s instructions. The potato *EF1α* gene was used as the internal reference for qPCR analysis [53,54]. The reaction system (10 μL) consisted of PrimeSTAR^®^ Max DNA Polymerase of 5 μL, primers (10mM) of 1 μL, and ddH_2_O of 4 μL. The reaction conditions were as follows: 95 °C, 3 min; 95 °C, 5 s; 60 °C, 30 s; 45 cycles. The qPCR results were calculated by 2^−ΔΔCT^ method. Primers used for qPCR are listed in Supplementary Table S2.

### 4.6. Statistical Analysis

The tissue-specific expression of *StAAO* genes and their expression analysis under abiotic stress were both performed for three independent biological replicates with three technical repeats. Statistical significances based on one-way ANOVA analyses were determined with Prism 7.00 software (GraphPad). The data were not log-transformed. Asterisks in the figures denote the significant differences as * *p* < 0.05, ** *p* < 0.01, and *** *p* < 0.001.

## 5. Conclusions

In summary, this study provides comprehensive information about the *AAO* gene family in potato, including gene structure, chromosome locations, phylogenetic relationships, promoter cis-element analysis, and expression patterns under various treatments. The response of the *StAAO* genes to various abiotic stresses and stress-related hormones indicates that *StAAOs* were involved in the tolerance of potato to environmental stress. Among them, *StAAO4* may be involved in the regulation of potato’s response to abiotic stress by promoting ABA synthesis. Subsequently, we will proceed with the validation of the biological function of the *StAAO4* gene by stable transformation in model plants or potatoes, followed by a comprehensive investigation of its molecular mechanism. Therefore, *StAAO4* might be a potential target for molecular breeding or genetic editing.

## Data Availability

All data are available within the manuscript.

## Supplementary Materials

The following supporting information can be downloaded at: https://www.mdpi.com/article/10.3390/plants12223809/s1, Table S1: the basic molecular characteristic information of StAAO proteins; Table S2: the sequences of primers were used for *StAAOs* in qRT-PCR; Table S3: the sequences used to construct phylogenetic tree.
