# Supplementary material for: Characterization and Identification of Drought-Responsive ABA-Aldehyde Oxidase (AAO) Genes in Potato (Solanum tuberosum L.)
Source: Plants (Basel). 2023 Nov 9;12(22):3809. doi: 10.3390/plants12223809 (PMC10674669; doi:10.3390/plants12223809)
Supplement: Supplementary file 1 [file plants-12-03809-s001.zip › Suplementary table S2.pdf]

Table S2 The sequences of primers were used for *StAAOs* in qRT-PCR

Gene	Sequences
StAAO2	F: 5'-CACCTTTGGCACCTCTTTGGAATG-3'
	R: 5'- CGAGTAGGAGGCTTAGGAGACAAC-3'
StAAO4	F: 5'-GCAAAGACAAGCAGGACTTCAAGG-3'
	R: 5'-AAGGCAAAGAAGCAAGACCAGTTG-3′
StAAO6	F: 5'-CCACTGCTGATTCTCCACACCTAG-3'
	R: 5'-TCCGAAACCTACGACCCAGAAATC-3'
