# Supplementary material for: Characterization and Identification of Drought-Responsive ABA-Aldehyde Oxidase (AAO) Genes in Potato (Solanum tuberosum L.)
Source: Plants (Basel). 2023 Nov 9;12(22):3809. doi: 10.3390/plants12223809 (PMC10674669; doi:10.3390/plants12223809)
Supplement: Supplementary file 1 [file plants-12-03809-s001.zip › Suplementary table S3.pdf]

Table S3 The sequences used to construct phylogenetic tree.

>OsAAO1
MRLSSLLFLVCFFTVAMSQCAAAAKARHFRWEVSNMFWSPDCEEKVVIGINGQFPGPTIRAKAGDTIVVHLK
NGLHTEGVVIHWHGIRQIGTPWADGTASISQCAINPEETFTYRFVVDKPGTYFYHGHYGMQRAAGLYGSLIVD
VADGDEEPFKYDGEINLLLSDWYHESIYTQMVGLSSNPFRWIGEPQSLLINGRGQFNCSLAAAHTPGAKQCAA
AGNRHCAPVILPVLPNKTYRLRVASTTSLASLNLAVGNHKLTVVEADGNYVEPFAVDDIDIYSGDSYSVLLTTDQ
DTSANYWVSVGVRGRQPRTAPALAVLNYRPNRASRLPAAAPPATPAWDDFARSKAFTYRILGRAGVTPPPPATS
DRRIELLNTQNRMGGGHVKWSINNVSMVLPATPYLGSLKMGLRSALPSAARPSDTFGRGYDVMRPPANPNTT
VGDNVYVLAHNATVDVVLQNANALARNVSEVHPWHLHGHDFWVLGYGDGAFRGDAGDAAALNLRNPPLR
NTAVIFPYGWTAIRFVADNPGVWAFHCHIEPHLHMGMGVIFAEAVDRVSELPKAAVSCGATATALMAGAGGH
V*

>OsAAO2
MAAAVQLLVVAAAAAMAAACCAGMAAAAATVEVTWDVEYVLWAPDCQQRVMIGINGRFPGPNITARAGD
VISVTMNNKMHTEGVVIHWHGIRQFGTPWADGTASISQCAVNPGETFVYKFVADKPGTYFYHGHFGMQRA
AGLYGSLIVLDSPEQPEPFRHQYDDGGELPMMLLSDWWHQNVYAQAAGLDGKDRHFEWIGEPQTILINGRG
QFECTLGPARKSFEKLLNENVETCVDDQKMCSDQEKCLRRSECGPYCPRSQCAPVVFNVEQGKTYRLRIASTTS
LSLLNVKIQGHKMTVVEADGNHVEPFVVDDIDIYSGESYSVLLKADQKPASYWISVGVRGRHPKTVPALAILSYG
NGNAAPPPLQLPAGEPPVTPAWNDTQRSKAFTYSIRARKDTNRPPPAAADRQIVLLNTQNLMDGRYRWSINN
VSLTLPATPYLGAFHHGLQDSAFDASGEPPAAFPEDYDVMRPPANNATTASDRVFRLRHGGVVDVVLQNANM
LREEVSETHPWHLHGHDFWVLGYGDGRYDPAAHAAGLNAADPPLRNTAVVFPHGWTALRFVANNTGAWAF
HCHIEPHLHMGMGVVFVEGEDRMHELD VPKDAMACGLVARTAATPLTPATPLPPSPAPAP*

>OsAAO3
MAPPPAAAAALAACILAVAATLAGADDPYRFFTWNVTYGSINPLGSTPQQGILINGQFPGPRIDCVTNDNIIVN
VFNNLDEPFLLTWNGIKQRKNSWQDGVLGTNCPIPPGANYTYKFQAKDQIGTFVYFPSVAMHRAAGGFGAL
NVYQRPAIPVPYPPPAGDFTLLVGDWYKAGHKQLRQALDAGGGGALPPPDALLINGMPSAAAFVGDQGRTYL
FRVSNVGVKTSVNVRIQGHSLRLVEVEGTHPVQNVYDSLDVHVGQSVAFLVTLDKAAQDYAVVASARFSPGAS
PLMATGTLHYSSAVSRAPGPLPAPPPEQAEWSMNQARSFRWNLTASAARPNPQGSFHYGTIATSRTLVLANSA
PVLAGQRRYAVNGVSFVVPDTPLKLVDNYNIANVIGWDSVPARPDGAAPRSGTPVVRLNLHEFIEVVFQNTEN
ELQSWHLDGYDFWVVGYGNGQWTENQRTTYNLVDAQARHTVQVYPNGWSAILVSLDNQGMWNLRSAN
WDRQYLGQQLYMRVWTPQQSFSNEYSIPTNAILCGRA AGLGH*

>OsAAO4
MMRCSDRLLCSLFLAAALFGVAAAATRRHDWDISYQFTSPDCVRKLAVTINGHTPGPTIRAVQGDTIVVNVKN
SLLTENVAIHWHGIRQIGTPWADGTEGVTQCPILPGDTFAYTFVVDRPGTYMYHAHYGMQRSAGLNGMIVVE
VAPGAAGDGEREPFRYDGEHTVLLNDWWHRSTYEQAAGLASVPMVWVGEPQSLLINGRGRFVNCSSSPATA
ASCNVSHPDCAPAVFAVVPGKTYRFRVASVTSLSALNFEIEGHEMTVVEADGHYVKPFVVKNLNIYSGETYSVLI
TADQDPNRNYWLASNVVSRKPATPTGTAVLAYYGGRRNSPRARPPTPPPAGPAWNDTAYRVRQSLATVAHPA
HAVPPPPTSDRTILLLNTQNKIGGQIKWALNNVSFTLPHTPYLVAMKRGLLGAFDQRPPPETYAGAAAFDVYAV
QGNPNATTSDAPYRLRFGSVVDVVLQNANMLAANSSETHPWHLHGHDFWVLGHGAGRFDPAVHPAAYNLR
DPIMKNTVAVHPFGWTALRFRADNPGVWAFHCHIEAHFFMGMGIVFEEGVERVGELPPEIMGCGKTRGGH*

>OsAAO5
MRTWRLAVLACLCAAAAAAPAEAKTHHHTWNITYQYKSPDCFRKLAVTINGESPGPTIRAAQGDTLVVTVHN
MLDTENTAIHWHGIRQIGSPWADGTAGVTQCPILPGETFTYRFVVDRPGTYMYHAHYGMQRVAGLDGMLVV
SVPDGVAEPFAYDGEHTVLLMDWWHQSVYEQAVGLASVPMVFVGEPQSLLINGRGVFNCSPPAASNGGGA
ACNAFGGECGWPTLFTASPGKTYRLRIGSLTSLASLSFEIEGHTMTVVEADGYYVTPVVVKNLFIYSGETYSVLVT
ADQDPSRSYWAASHVVSRDPTKTAPGRAVVRYASAAVDHPRTPPPTGPRWNDTASRVAQSRSFAALPGHVE
PPPARPDRVLLLLNTQSKIDNHTKWAINGVSLSFPATPYLVAMKHGLRGEFDQRPPPDSYDHGSLNLSSPPASLA
VRHAAYRLALGSVVDVVLQNTAIPPPNGRSETHPWHLHGHDFWVLGYGEGKFVPEVDGPGLNAASARGGAV
MKNTVALHPMGWTAVRFRASNPGVWLFHCHLEAH VYMGMGVVFEEGVDVLPRLPASIMGCGRTKGHHY*

>AtAAO1
MMRPKRSSDTVHVFNLMVLCFIALFFSSVLCQGKIRRFKWEVKYEFKSPDCFEKLVITINGKFPGPTIKAQQGDT
IVVELKNSFMTENVAVHWHGIRQIGTPWFDGVEGVTQCPILPGEVFIYQFVVDRPGTYMYHSHYGMQRESGL
IGMIQVSPPATEPEPFTYDYDRNFLLTDWYHKSMSEKATGLASIPFKWVGEPQSLMIQGRGRFNCSNNLTTPPS
LVSGVCNVSNADCSRFILTVIPGKTYRLRIGSLTALSALSFQIEGHNLTVVEADGHYVEPFTVKNLFVYSGETYSVLL
KADQNPRRNYWITSSIVSRPATTPPATAVLNYYPNHPRRRPPTSESSNIVPEWNDTRSRLAQSLAIKARRGFIHAL
PENSDKVIVLLNTQNEVNGYRRWSVNNVSYHHPKTPYLIALKQNLTNAFDWRFTAPENYDSRNYDIFAKPLNA
NATTSDGIYRLRFNSTVDVILQNANTMNANNSETHPWHLHGHDFWVLGYGEGKFNESEDPKRYNRVDPIKK
NTVAVQPFGWTALRFRADNPGVWSFHCHIESHFFMGMGIVFESGIDKVSSLPSSIMGCGQTKR

>AtAAO2
MAVIVWWLLTVVVVAFHSASAAVVESTWEVEYKYWWPDCKEGIVMAINGQFPGPTIDAVAGDTVIIHVVNKL
STEGVVIHWHGIRQKGTPWADGAAGVTQCPINPGETFTYKFIVDKAGTHFYHGHYGMQRSSGLYGMLIVRSP
KERLIYDGEFNLLLSDWWHQSIHAQELALSSRPMRWIGEPQSLLINGRGQFNCSQAAYFNKGGEKDVCTFKEN
DQCAPQTLRVEPNRVYRLRIASTTALASLNLAVQGHQLVVVEADGNYVAPFTVNDIDVYSGETYSVLLKTNALPS
KKYWISVGVRGREPKTPQALTVINYVDATESRPSHPPPVTPIWNDTDRSKSFSKKIFAAKGYPKPPEKSHDQLILL
NTQNLYEDYTKWSINNVSLSVPVTPYLGSIRYGLKSAYDLKSPAKKLIMDNYDIMKPPPNPNTTKGSGIYNFAFGI
VVDVILQNANVLKGVISEIHPWHIHGHDFWVLGYGEGKFKPGIDEKTFNLKNPPLRNTVVLYPFGWTAIRFVTD
NPGVWFFHCHIEPHLHMGMGVVFVEGVDRIGKMEIPDEALGCGLTRKWLMNRGRP

>AtAAO3
MSYDEHTSSSFTYISQMGVWWIVLVVAVLTHTASAAVREYHWEVEYKYWSPDCKEGAVMTVNGEFPGPTIKA
FAGDTIVVNLTNKLTTEGLVIHWHGIRQFGSPWADGAAGVTQCAINPGETFTYNFTVEKPGTHFYHGHYGMQ
RSAGLYGSLIVDVAKGKSERLRYDGEFNLLLSDWWHEAIPSQELGLSSKPMRWIGEAQSILINGRGQFNCSLAA
QFSNNTSLPMCTFKEGDQCAPQILHVEPNKTYRIRLSSTTALASLNLAVQGHKLVVVEADGNYITPFTTDDIDIYS
GESYSVLLTTDQDPSQNYYISVGVRGRKPNTTQALTILNYVTAPASKLPSSPPPVTPRWDDFERSKNFSKKIFSAM
GSPSPPKKYRKRLILLNTQNLIDGYTKWAINNVSLVTPATPYLGSVKYNLKLGFNRKSPPRSYRMDYDIMNPPPFP
NTTTGNGIYVFPFNVTVDVIIQNANVLKGIVSEIHPWHLHGHDFWVLGYGDGKFKPGIDEKTYNLKNPPLRNTA
ILYPYGWTAIRFVTDNPGVWFFHCHIEPHLHMGMGVVFAEGLNRIGKVPDEALGCGLTKQFLMNRNRN

>NtAAO1
MASLGFLFFFLLPLILLELSSSRSVMAAKTRHFKWDVEYIHWSPDGEESVVMGINGQFPGPTIRAKAGDTVAVH
LTNKLHTEGVVIHWHGIRQIGTPWADGTAAISQCAINPGETFLYRFKVDKAGTYFYHGHYGMQRSAGLYGSLIV
EVGEGEKEPFHYDGEFNLLLSDWWHKGSHEQEVDLSSNPLRWIGEPQTLLLNGRGQYNCSLAARFSKPPLPQC

KLRGGEQYAPQILRVRPNKIYRLRVASTTALGSLSLAIGGHKMVVVEADGNYVQPFSVQDMDIYSGESYSVLFKT
DQDPTKNYWISINVRGREPKTPQGLTLLNYLPNSASKFPTLPPPIAPLWNDYNHSKSFSNKIFALMGSPKPPPQ
NHRRIILLNTQNKIDGYTKWAINNVSLVLPTQLYLGSIRYGINAFDTKPPPDNFPKDYDVLKQAPNSNSTYGNGV
YMLKFNTTIDIILQNANALAKDVSEIHPWHLHGHDFWVLGYGEGKFSEKDVKKFNLKNPPLRNTAVIFPFGWTA
LRFVTDNPGVWAFHCHIEPHLHMGMGVIFAEGVHLVKKIPKEALACGLTGKMLMSNKHN

>NtAAO2
MGSGKVTFVALLLCLSVGVIAEDPYLYFNWNVTYGTIAPLGVPQQGILINGQFPGPRINCTSNNNIVVNVFNNL
DEPFLFTWNGVQHRKNSWQDGTPGTMCPIMPGQNFTYRFQVKDQIGSYSYFPTTALHRAAGGYGALNVHS
RALIPVPFDNPADEYNVFVGDWYNKGHKTLKKILDGGRTIGRPDGIIINGKSAKVGEAKEPLFTMEAGKTYRYRF
CNLGMRSSVNIRFQGHPMKLVELEGSHTVQNIYDSLDLHVGQCLSVLVTADQEPKDYYLVVSSRFLKQALSSVAI
IRYANGKGPASPELPTPPPENTEGIAWSMNQFRSFRWNLTASAARPNPQGSYHYGQINITRTIKIFNSMSQVGG
KLRYGLNGISHTNGETPLKLVEYFGATNKAFKYDLMADEAPADPSKLTIATNVKNATYRNFVEIIFENHEKTIRTYH
LDGYSFFAVAVEPGRWSPEKRKNYNLVDGLSRNNIQVYPNSWAAIMLTFDNAGMWNLRSEMWEKTYLGEQL
YFSVLSPSRSLRDEYNIPDNHPLCGIVKGLSMPAPYKA

>GmAAO1
MVELRLSIRALQLPTLLILCFFLILGNFHKAEARIRHHKWEVKYEFRSPDCFKKLVITINGKTPGPTIQAQEGDTIVV
QVNNSLVTENLSIHWHGIRQIGTPWFDGTEGVTQCPILPGDTFIYQFVVDRPGTYLYHAHYGMQREAGLYGMI
RVAPRDPEPFAYDLDRSIILNDWYHKSTYEQAAGLSSIPFQWVGEPQSLLIHGKGRFNCSKSPSVSTDVCDTSNP
QCSPFVQTVIPGKTYRLRIASLTALSALSFEIEANDMTVVEADGHYVEPFEVKNLFIYSGETYSVLVKTDQDPSRN
YWITSNVVSRNRTTPPGLGMFNYYPNHPKRSPPTVPPSPPAWDDVEPRLAQSLSIKARQGYILKPPTTSDRVIVL
LNTQNNISEYRHWSVNNVSFTLPHTPYLISLKENITGAFDPTPPPDGYDFANYDIFSVASNANATSSSGIYRLKFN
TTVDIILQNANTMNKNNSETHPWHLHGHDFWVLGYGKGKFDVNNDTKKYNLENPIMKNTVPVHPFGWTAL
RFRTDNPGVWAFHCHIESHFYMGMGVVFEEGIERVGKLPSSIMGCGQTRGFHRP

>GmAAO2
MVELGLISLRALQLPRLLILCFFVILGNFHKAEARIRHYKWEAKYEFRSPDCFKKLVITINGKTPGPSIQAQEGDTII
VQVNNSLVTENLSIHWHGIRQIGTPWFDGTEGVTQCPILPGDTFIYQFVVDRPGTYLYHAHYGIQREAGLYGM
MRVAPRDPEPFAYDLDRSIILNDWYHSSTYEQAAGLSSIPFRWVGEPQSLLIHGKGIFNCSKSPSLGTDVCDASK
CSPFVQTVIPGKTYRLRIASLTALSALSFQIEGHNMTVVEADGHYVEPFVVKNLFIYSGETYSVTVKSDQDPSRNY
WITSNVVSRNRSTPAGLGMFNYYPNHPKRSPPTVPPSPPAWHDVEPRLAQSFSIKARQGYIHKPPTTSDRVIVL
LNTQNNISEYRHWSVNNVSFTLPHTPYLIALKENINGAFDSTPPPDGYDFANYDIFSVASNANATSSSGIYRLKFN
TTVDIILQNANTMTKTNSETHPWHLHGHDFWVLGYGKGKFDVNNDTKKYNLENPIMKNTVPVHPFGWTALR
FRTDNPGVWAFHCHIESHFYMGMGVVFEEGVERVGKLPSSIMGCGQTRGFHGP

>GmAAO3
MVTMGLKALFVWCIIWLAFAQLSLGGRVRHYKFDVEYMIRKPDCLEHVVMGINGQFPGPTIRAEVGDILDIAL
TNKLFTEGTVIHWHGIRQVGTPWADGTAAISQCAINPGETFQYRFTVDRPGTYFYHGHHGMQRSAGLYGSLIV
DLPKGQNEPFPYDGEFNLLLSDLWHTSSHEQEVGLSSKPFKWIGEAQTLLINGRGQFNCSLASKFINTTLPQCQL
KGGEECAPQILHVEPNKTYRIRIASTTALASLNLAISNHKLVVVEADGNYVTPFAVDDIDIYSGESYSVLLRTDQDP
NKNYWLSIGVRGRKPNTPQGLTILNYKPISASVFPTFPPPITPLWNDFERSKAFTKKIIAKMGTPQPPKRSDRTIFL
LNTQNRVDGFTKWAINNVSLTLPPTPYLGSIKFKIKNAFDKTPPPVTFPQDYDIFNPPVNPNASIGNGVYMFNL
NEVVDVILQNANQLSGSGSEIHPWHLHGHDFWILGYGEGKFKSGDEKKFNLTHAPLRNTAVIFPYGWTALRFK

ADNPGVWAFHCHIEPHLHMGMGVIFAEAVQKVGKIPRDALTCGLTGKMLGNRHY

>GmAAO4
MSKSNNNIMSFKALTLWCILLSLLQLSLGAVRHRIRFNVEYMYREPDCHEHVVMGINGQFPGPTITAEAGDTLEI
LLTNKLSTEGTVIHWHGIRQYGTPWADGTAAISQCAIAPGETFNYTFTVDRPGTYFYHGHFGMQRAAGLYGSLI
VNLPKGKKEPFHYDGEFNLLLSDWWHKSTHSQEVGLSSMPFRWINEPQSLLINGRGQYNCSLAASLIKTSLPQ
CKFRGNEQCAPQILHVDPNKTYRIRIASTTSLASLNLAIGDHKLVVVEADGNYVKPFIVDDIDIYSGESYSVLLTTN
QDPKKNYWISVGVRGRPPNTPQGLTILNYKTISASVFPTSPPPITPQWDDYNRSKAFTYKILALKGTEQPPQHYD
RRLFLLNTQNLVDGYTKWAINNVSLALPTTPYLGSIRFNVNGAFDPKSPPDNFSMDYDILKPPLNPNAKIGSGVY
MFQFNQVVDVILQNANVMKGKNSEIHPWHLHGHDFWILGYGDGKFKQGDDSKFNLKNPPLRNTAVIFPHG
WTALRFKADNPGVWAFHCHIEPHLHMGMGVIFAEAVQNVTSTIPRDAFACGILKKFLNKEHN

>GmAAO5
MGLKALFVWCIIWLGLAHLSLGGRVRHYKFDVEYMIRKPDCLEHVVMGINGQFPGPTIRAEVGDILDIALTNKL
FTEGTVIHWHGIRQVGTPWADGTAAISQCAINPGEAFHYRFTVDRPGTYFYHGHHGMQRSAGLYGSLIVDLPK
GQNEPFHYDGEFNLLLSDLWHTSSHEQEVGLSSKPFKWIGEPQTLLINGKGQFNCSLASKFINTTLPQCQLKGG
EECAPQILHVEPNKTYRIRIASTTALASLNLAISNHKLVVVEADGNYVSPFAVDDIDIYSGESYSVLLRTDQDPNKN
YWLSIGVRGRRAPNTPQGLTILNYKPISASIFPISPPPITPIWNDFERSKAFTKKIIAKMGTPQPPKRSDRTIFLLNT
QNLLDGFTKWAINNVSLTLPPTPYLGSIKFKINNAFDKTPPPVTFPQDYDIFNPPVNPNTTIGNGVYMFNLNEV
VDVILQNANQLSGSGSEIHPWHLHGHDFWVLGYGEGKFKPSDEKKFNLTHAPLRNTAVIFPYGWTALRFKADN
PGVWAFHCHIEPHLHMGMGVIFAEGVHKVGKIPRDALTCGLTGNKLVGNRHY

>GmAAO6
MSLKALFVGCIIWLGLVELSIGGIVRHYKFDVEYMIRKPDCLEHVLMGINGQFPGPTIRAEVGDILDIALTNKLFTE
GTVIHWHGIRQVGTPWADGTASISQCAINPGETFHYKFTVDRPGTYFYHGHHGMQRAAGLYGSLIVDLPKGQ
NEPFHYDGEFNLLFSDLWHTSSHEQEVGLSTKPLKWIGEPQTLLINGRGQFNCSLASKFINTTLPECQFKGGEEC
APQILHVEPNKTYRIRIASTTSLAALNLAISNHKLVVVEADGNYVTPFAVDDVDIYSGESYSVLLRTDQDPNKNY
WLSIGVRGRKPSTSQGLTILNYKTISASIFPTSPPPITPLWNDFEHSKAFTKKIIAKMGTPQPPKLYDRRVFLLNTQ
NRVDGFTKWSINNVSLTLPPTPYLGSIKFKINNAFDQTPPPMNFPQDYDIFNPPVNPNATIGNGVYMFNLNEV
VDVILQNSNQLSVNGSEIHPWHLHGHDFWVLGYGEGKFKLGDEKKFNLTHAPLRNTAVIFPYGWTALRFKADN
PGVWAFHCHIEPHLHMGMGVIFAEGVHKVGKIPREALTCGLTGKKLVENGHY

>GmAAO7
MSLKALFVWCIIWLGLVELSLGGRVRHYKFDVEYMIRKPDCLEHVVMGINGQFPGPTIRAEVGDILDIALTNKLF
SEGTVVHWHGIRQVGTPWADGTASISQCAINPGETYHYRFTVDRPGTYFYHGHYGMQRAAGLDKTNRFHYD
GEFNLLLSDLWHTSSHEQEVGLSTKPLKWIGEPQTLLINGRGQFNCSLASKFINTTLPQCHLKGDEECAPQILDV
EPNKTYRIRIASTTSLAALNLAISNHKLVVVEVDGNYVTPFAVDDMDIYSGESYSVLLHTNQNPNKNYWLSIGVR
GRKPNTPQGLAILNYKTISALIFPTSPPPITPLWNDFEHSKAFTKKIIAKMGTPQPPEHSDRTQYSSSTPKIELMDY
HIFNPPVNPNATIGNGVYMFNLNEVVDVILQNANQLIGNGSEIHPWHLHGHDFWVLGYGEGKFKSGDVKKF
NFTQAPLRNTAVIFPYGWTALRFKADNPGVWAFHCHIEPHLHMGMGVVFAEGVHKVGKIPREALTCGLTGKKL
IENGRY

>ZmAAO1
TGITAPLESRHIAAACLCTRAQSGTPISHIIGSSSCAAPHCVDPAMGCPPRLLCCLFLSLSLAAVVARAATRHQEW

EISYQFKSPDCVRKLAVTINGETPGPTIRATQGDTVVVRVKNSLLTENVAIHWHGIRQRGTPWADGTEGVTQCP
ILPGDTFTYAFVVDRPGTYMYHAHYGMQRSAGLNGLIVVAAAPGGPDAEPFRYDGEHDVLLNDWWHKSTYE
QAAGLASAPLVWVGEPQSLLINGRGRFVNCSAAGACDAAHPECATPVFAVVPGRTYRFRIASVTSLSALNFEIE
GHEMTVVEADGHYVKPFVVKNLNIYSGETYSVLIKADQDPNRNYWLASNVVSREPGTPAGTAVLSYYGGRSSP
RRSPPTAPPAGPAWNDTAYRFRQSVATVAHPAHVEPPPPRADRTILLLNTQNKIDAHIKWALNGVSFTLPHTPYL
VAMKRPGLLDTFDQRPPPETYAHRGYDVYAVPPNPNATTSDGLYRLRFGSVVDVVLQNANMLAPNKSETHP
WHLHGHDFWVLGYGIGRFDPAVHPASYNLRDPILKNTVAVHPYGWTALRFRADNPGVWAFHCHIESHFFMG
MGIAFEEGVDRVAPLPPQIMGCGKTRGGH

>ZmAAO2
MALLPGAARRLLCSLFLCLWLSTLARAATRRYEWEVSYQFKSPDCVRKLSATINGQTPGPTIRATQGDTVEVKVR
NSLLTENLAIHWHGIRQIGTPWADGTEGVTQCPILPGDTLTYAFVVDRPGTYMYHAHYGMQRSAGLYGVVVV
VVAAAPGAKADDAEPFAYDDEHHVLLNDWWHNSTYELAVGLASVPMVWVGEPHSLLINGRGRFNCSAAAV
PGTCNATSPECDTPVFAVVPGQTYRFRIASVTSLSALNFEIEGHEMTVVEADGHYVKPFVVKNINIYSGETYSVLI
KADQDPNRNYWLASNVVSRQPATPTGTAILSYSGDRAPPSTTPPTGPAWNDTMYRFQQSVATVAHPAYVEPP
PPRADRTILLLNTQNKIDAHTKWALNGVSFTLPHTPYLVAMKRGLLDTFDQRPPPETYAYQGYDVYAPPQNPNA
TTSDGLYRLRFGSVVDVVLQNANMLAPNKSETHPWHLHGHDFWVLGYGIGRFDPAVHPASYNLKDPVLKNTV
AVHPYGWTALRFKADNPGVWAFHCHIEAHFFMGMGIVFEEGIQRVASLPPEIMGCGETNGGHR

>ZmAAO3
ARRSFSSRSGLTMSAPRQPLCKLATAVHHLLLCSLAALSVSADAKVHHHTWDIAYHYKSLDCVNKLAVTINGESP
GPTIRATQGDTVVVTVRNSLETENTGIHWHGIRQVGSPWADGTVGVTQCPILPGDTFTYRFVVDRPGTYFYHA
HYGMQRVAGLDGMLVVSVPDGVAEPFAYDEDRTVLLMDWWHKSVYEQAVGLASDPLVFVGEPQSLLINGRG
VFEPFHCSRAPSASGCSSAPRPAAGCAPPALFTAVPGKTYRLRVGSLTSLSALNARERNTTSAMAILSYAGNDPR
APPPTPRPEGPAWDDARPRLEQSRSLAVAHPDHVVPVPPRPDRALLLLNTQNRIGGHIRWAINGVSLAFPATPY
LVSIKRGLRGAYDDQRPPPDTYDYRSYDIASPPTANGTVASKVYRLALGSVVDVVLQNTVALNNKSETHPWHLH
GHDFWVLAYGDDGKKFDPERDTNKFNLRDPVMKNTVALHPRGWTAVRFVADNPGVWLFHCHIEAHVYMG
MGLVFEEGVDKVGRLPKSIMGCGRSRT

>ZmAAO4
MRLPLPLLALVCCALMARQHCAAAGKARHLRWEISNMFWSPDCEEKVVIGINGQFPGPTIRARAGDTVHVQL
RNALHTEGVVIHWHGIRQIGTPWADGTAAISQCAINPEETFTYRFVVDKPGTYFYHGHYGMQRAAGLYGSLVV
DVAEGEEEPFQYDGELNLLLSDWYHESIHTQMVALSSRPFRWIGEPQSLLINGRGQFNCSLAAAHTQGATNTQ
CAATAANTQCAPVVLPVQPNKTYRLRVASTTSLASLNLAIGNHKLTVVEADGNYVDPFVVDDIDLYSGDSYSVLL
TTDQDTSSNYWVSVGVRGRLPKTAPALAVLNYRPNRASDLPALAPPVTPAWDDYGHSKAFTYRIRARAGTPPP
PPTAARRIELLNTQNRMDGRIRWSINNVSMVLPATPYLGSLKMGLKSTLAAARPAETFSREYDVTLPPPNPNTT
AGDNVYVLAHNTTVDVLLQNANALSRNVSEVHPWHLHGHDFWVLGYGDGAYRGDAADEARLNLRDPPLRN
TAVIFPYGWTMLRFVADNPGVWAFHCHIEPHLHMGMGVIFAEAVDLVAKVPNEAVSCGATATALMAGGHL

>TaAAO1
MSGGGAAMESSLLCAKQLLLCCLFLFLWALAAVAEAKTVHEQWDISYQFTHSDCVRKLAVTINGGTPGPTIRAV
QGDTVVVTVKNLLMTENVAIHWHGIRQLGTPWADGTEGVTQCPILPGDTFEYRFVVDRPGTYMYHAHYGM
QRSAGLNGMIVVAAAPGSADAEPFAYDGGEHEVLLNDWWHKSTYEQAAGLAAVPIVWVGEPQSLLINGRGR
YNCSAMASDAAAACNATHPECAPQVFAVVPGRTYRFRIASVTSLSALNFEIEGHEMTVVETDGHYVKPFVVKN

LNVYSGETYSVLIKADQDPNRNYWLASNVVSRKPGTPTGTAVLSYYGGRSSPRAPPPTAPPAGPAWNDSAYRIN
QSLATVAHPEHAHPPPPRADRTILLLNSQNKIDGRIKWAINNVSFTLPHTPYLVALKHGLLGAFDQRPPPETYNH
TGYDVYGVQANPNATTSDGLYRLAFGSVVDVVLQNANMLAPNNSETHPWHLHGHDFWTLGFGVGRFDPAV
HPATYNLRDPVMKNTVAVHPFGWTALRFRADNPGVWAFHCHIEAHFFMGMGVAFEEGIERVGDLPEEIRRC
VSTKGGGH

>TaAAO2
MLPSARPLLAAAAVLCFWLLVAVAEAKVHHYTWDISYQLKSPDCFEKLAVTVNGEAPGPTIRATQGDTIVVAVH
NKLETENTAIHWHGIRQIDTPWADGVAGVTQCPILPGETFTYQFVVDRPGTYLYHAHYGMQRVAGLNGMIVV
TVPDGFVEPFSYDEEHTVLLGDWWHKSVYEQATGLSSNPFVFVTEPQSLLINGRGMFNCSLAPSGTCNASRPD
CALPTLFTAVPGKTYLLRIGSLTSLSSLYFEIEGHSMMVVEADGHYVLPFAVRGLFIYSGETYSVLVKADQDPRRNY
WAASHVVGRNPSQTPSGKAVVSYAFNGNNPWMPPPTAPPAGRAWNNTAIRVEQSRAIFAHPRYVVPMPAR
ADRTLLLLNTQNRIDGHIKWTINGVSLMFPATPYLVAMKRGMKDAYEQRPPPDMYDHMSHEISAPAPTNGTV
GSPVYRLALGSVVDVVLQNSNALNNKTETHPWHLHGHDFWVLGHGEGKFNPAADAWRLLNVRDPIMKNTV
PLHPDGWTAVRFRADNPGVWLFHCHVEAHVFMGMGVVFEEGVKRVGRLPSSIMGCGRSKGLH

>TaAAO3
MESSLLGAKQLLLCCLFLLWVLAAVAEAKTVHEQWEISYQFTHSDCVRKLAVTINGGTPGPTIRAVQGDTVVVT
VKNLLMTENVAIHWHGIRQLGTPWADGTEGVTQCPILPGDTFEYRFVVDRPGTYMYHAHYGMQRSAGLNG
MIVVAAAPGGADAEPFAYDGGEHDVLLNDWWHKSTYEQAAGLAAVPIVWVGEPQSLLINGRGRYNCSAMA
PDAAACNATHPECAAQVFAVVPGRTYRFRIASVTSLSALNFEIEGHEMTVVETDGHYVKPFVVKNLNIYSGETYS
VLIKADQDPNRNYWLASNVVSRKPGTPTGTAVLSYYGGRSSPRAPPPTAPPAGPAWNDSAYRIGQSLATVAHP
EHAHPPPPRADRTILLLNSQNKIDGRIKWAINNVSFTLPHTPYLVALKHGLLGDFDQRPPPETYNHTGYDVYGV
QANPNATTSDGLYRLAFGSVVDVVLQNANMLAPNNSETHPWHLHGHDFWTLGYGVGRFDPAVHPATYNLR
DPVMKNTVAVHPFGWTALRFRADNPGVWAFHCHIEAHFFMGMGVAFEEGIERVGDLPEEIRRCVSTKGGGH

>TaAAO4
MLPSARPLLAAAAVLCFWLLVAVAEAKVHHYTWDISYQLKSPDCFEKLAVTVNGEAPGPTIRATLGDTIIVAVHN
KLETENTAIHWHGIRQIDTPWADGVAGVTQCPILPGETFTYKFVVDRPGTYLYHAHYGMQRVAGLNGMIVVT
VPDGFVEPFSYDEEHTVLLGDWWHKSVYEQATGLSSNPFVFVTEPQSLLINGRGMFNCSLAPSGTCNASRPDC
ALPTLFTAVPGKTYLLRIGSLTSLSSLYFEIEGHSMMVVEADGHYVRPFAVRGLFIYSGETYSVLVKADQDPRRNY
WAASHVVGRNPSQTPTGKAVVSYAFNGNNPWMPPPTAPPAGPPWNNTAIRVEQSRAIFAHPRFVEPMPAR
ADRTLLLLNTQNRIDGHIKWTINGVSLMFPATPYLVAMKRGMKDAYEQRPPPDMYDHMSHDISAPAPTNGTV
GSPVYRLALGSVVDVVLQNSNMLNNKSETHPWHLHGHDFWVLGHGEGKFNPAADAWRLLNVRDPIMKNT
VPLHPDGWTAVRFRADNPGVWLFHCHVEAHVFMGMGVVFEEGVERVGRLPSSIMGCGRSKGLH

>TaAAO5
MRGGGAMESSLLGANPRLLCCLFLLWALAAVAEAKTVHKQWDISYQFTHSDCVRKLAVTINGRTPGPTIRAVQ
GDTVVVTVKNLLMTENVAIHWHGIRQLGTPWADGTEGVTQCPILPGDAFEYRFVVDRPGTYMYHAHYGMQ
RSAGLNGMIVVAAAPGSADAEPFAYDGGEHEVLLNDWWHKSTYEQAAGLAAVPIVWVGEPQSLLINGRGRY
NCSAMAPDAACNATHPECAAQVFAVVPGRTYRFRIASVTSLSALNFEIEGHEMTVVETDGHYVKPFVVKNLNI
YSGETYSVLIKADQDPNRNYWLASNVVSRKPGTPTGTAVLSYYGGRSSPRAPPPTAPPAGPAWNDSAYRIRQSL
ATVAHPEHAHPPPPRADRTILLLNSQNKIDGRIKWAINNVSFTLPHTPYLVALKHGLLGAFDQCPPPETYNHTGY
DVYGVQANPNATTSDGLYRLAFGSVVDVVLQNANMLAPNNSETHPWHLHGHDFWTLGYGVGRFDPAVHPA

TYNLRDPVMKNTVAVHPFGWTALRFRADNPGVWAFHCHIEAHFFMGMGVAFEEGIERVGDLPEEIRRCVSTK
GGHH

>TaAAO6
MLPSARPLLAPAAAVLCFWLLVAVAEAKVHHYKWDISYQLKSPDCFEKLAVTVNGEAPGPTIRATLGDTIVVDV
HNKLETENTAIHWHGIRQIGTPWADGVAGVTQCPILPGETFTYKFVIDRPGTYLYHAHYGMQRVAGLNGMIV
VTVPEGFVEPFSYDEEHTVLLGDWWHKSVYEQATGLSANPFVFVTEPQSLLINGRGMFNCSLAPSGTCNASRP
DCALPTLFTAVPGKTYLLRIGSLTSLSSLYFEIEGHPMMVVEADGHYVRPFAVRGLFIYSGETYSVLVKADQDPRR
NYWAASHVVGRNPSQTPSGKAVVSYAFNGNNPWMPPPTAPPAGPPWNNTAIRVDQSRAIFAHPHFVEPMP
ARADRTLLFLNTQNRIDGHIKWTINGVSLMFPATPYLVAMKRGMKDAYEQRPPPDMYDHMSHDISAPAPTNG
TVGSPVYRLALGSVVDVVLQNSNMLNNKSETHPWHLHGHDFWVLGYGEGKFNPAADAWRLLNVRDPIMKN
TVPLHPDGWTAVRFRADNPGVWLFHCHVEAHVFMGMGVVFEEGVERVGRLPSSIMGCGRSKGLH

>TaAAO7
MTRPHTGSEPPLVMHLLLCCTFLLAFAAPATTAASAPAPAPTPAKQTMTWDVEYIMWAPDCQQRVMIGINGK
FPGPNITARAGETLSITVNNKLHTEGLVIHWHGMRQVGTPWADGTASISQCAVSPGDSFTYEFVADKPGTYFY
HGHFGMQRAAGLYGWLVVDATAERGEPYRRDYDGGELRMLLSDWYHDNVYAQAAGLEQKYDHFQWVGEP
QTILVNGRGQYDCMLGAVTRFHRGIDRRARTCVRGKEAKLCGDEERCLRRSECGPYCPESQCAPVVLDVEPGR
TYRLRIASTTSLAALNVQVQGHELTVVEADGNPVEPFTVADIDIYSGESYSVLLTTNHTPTFYRSGSFWVSVGVR
GRPPKTLPATAVLRYTNSRFPWPGSPPPATPAWYDLQRSKDFARRIKARRNAAEAPPPPRTEQVSRRIVMLNTQ
TLVDGHIKWAVNNVSLTLPTTPYLGAYFYGVQGSAFDASGEAPNGFPGGYDIDLPPENNSYEATLSDRVYELAH
GAVVDVVLQNADMRRDNDSETHPWHLHGHDFWVLGYGEGRYTGGERLNTEDPPLRNTVVVFPHGWTAIRF
VADNVGAWAFHCHIEPHLHMGMGAVFVEGAHMIRELDVPREAMMCGVIRTTVAALTPAKPGSPAPAP

>TaAAO8
MRALFAWCALLLACGGLLHCAEAAKARHLKWEVGHMFWSPDCEEKVLIGINGQFPGPTIRAKAGDTIVVELK
NGLHTEGVVIHWHGVRQIGTPWADGTAAISQCAINPEETFTYRFVVDKPGTYFYHGHYGMQRAAGLYGSLIV
DVADGEEEPFKYDGELNLLLSDWYHDSIYNQMVGLSSSPMRWIGEPQSLLINGRGQFNCSLAAAHTPGTKQC
TAGGNRHCAPVILPVQPNKTYRLRIASTTSLASLNLAIGNHKLTVVEADGNYVEPFVVDDMDIYSVTPAWNDTA
HSKAFTTQIKARAGTPPPPATSDRRIELLNTQNKLDGHIKWSINNVSLVLPATPYLGSLKLGLKTALAAARPADTFG
RAYDVTRPPHNPNTTTGDNVYVLRHNTTVDVVLQNANALQHNVSEVHPWHLHGHDFWVLGYGEGAYRGD
AADAARLNLANPPLRNTAVIFPYGWTALRFVADNPGVWAFHCHIEPHLHMGMGVIFAEAIDRVGKVPKEAVS
CGATATALMNGDHL

>TaAAO9
MRALFAWCALLLACAGVMHCTEAAKARHLKWEVSHMFWSPDCEEKVLIGINGQFPGPTIRAKAGDTIVVELK
NGLHTEGVVIHWHGVRQIGTPWADGTAAISQCAINPEETFTYRFVVDKPGTYFYHGHYGMQRAAGLYGSLIV
DVADGEEEPFKYDGELNLLLSDWYHDSIYNQMVGLSSSPMRWIGEPQSLLINGRGQFNCSLAAAHTPGTKQC
TAGGNRHCAPVILPVEPNKTYRLRIASTTSLASLNLAIGNHKLTVVEADGNYVEPFVVDDMDIYSGDSYSVLLTT
DQDPSSNYWVSIGVRGRTPKTAPALALLNYRPNRGFKLPAIAPPVTPLWNDTAHSKAFTTHIKARAGTPPPPATS
DRRIELLNTQNKLDGHIKWSINNVSMVLPATPYLGSLKLGLKTALTAARPADTFGRAYDVTRPPHNPNTTTGDN
VYVLRYNTTVDVVLQNANALQHNVSEVHPWHLHGHDFWVLGYGEGAYRGDAADAARLNLVNPPLRNTAVIF
PYGWTALRFVADNPGVWAFHCHIEPHLHMGMGVIFAEAIDRVGKVPKEAVSCGATATALMNGDHL

>TaAAO10
MTRPHTGSESPLVVHLLLCCTFLLAAAAPATTAASVPAPAPTPTKQNMTWDVEYIMWSPDCQQRVMIGINGK
FPGPNITARAGETLSITVNNKLHTEGLVIHWHGMRQVGTPWADGTASISQCAISPGDSFTYEFVADKPGTYFYH
GHFGMQRAAGLYGWLVVNATAEQDEPYRRDYDGGELRMLLSDWYHDNVYAQAAGLEQKYDHFQWVGEPQ
TILINGRGQHDCMLGTVTRFHRGIDRHAKTCVRDKQAKLCRDEERCLRRSECGPYCPQSQCYPVVFDVEPGRT
YRLRIASTTSLSALNVQVQGHDLTVVEADGNPVEPFTVPDIDIYSGESYSVLLTTNHTPTFYRSGSFWVSVGVRG
RPPKTLPATAVLRYTNSRFPWPGSPPPATPAWYDLQRSKGFTYRIKARRNAAEAPPPPRTEQVNRTIVMLNTQTL
VGGHMKWAVNNVSLTLPATPYLGAYFYGVQGSAFDASGEAPNGFPGGYDIDLPPANNSYEATLSDRVYELPHG
AVVDVVLQNADMRRDNDSETHPWHLHGHDFWVLGYGEGRYGGGGGGERLNTEDPPLRNTVVLFPHGWTA
IRFVADNVGAWAFHCHIEPHLHMGMGAVFVEGAHMIRELDVPRETMMCGVIRTAAASLTPAKPGSPAPSAH
G

>TaAAO11
MRALFAWCALLLACGGVMHCAEAAKARHLKWEVSHMFWSPDCEEKVLIGINGQFPGPTIRAKAGDTIVVELK
NGLHTEGVVIHWHGVRQIGTPWADGTAAISQCAINPEETFTYRFVVDKPGTYFYHGHYGMQRAAGLYGSLIV
DVADGEEEPFKYDGELNLLLSDWYHESIYNQMVGLSSSPMRWIGEPQSLLINGRGQFNCSLAAAHTPGTKQCT
AGGNRHCAPVILPVEPNKTYRLRIASTTSLASLNLAIGKHKLTVVEADGNYVAPFVVDDMDIYSGDSYSVLLTTD
QDPSSNYWVSIGVRGRTPKTAPALALLNYRPNRGFKLPAIAPPVTPAWNDTAHSKAFTTQIKARAGTPPPPATS
DRRIELLNTQNKLDGHIKWSINNVSLVLPATPYLGSLKLGLKTALAAARPADTFGRAYDVTRPPHNPNTTTGDNV
YVLRHNTTVDVVLQNANALQHNVSEVHPWHLHGHDFWVLGYGEGAYRGDAADAARLNLVNPPLRNTAVIFP
YGWTALRFVADNPGVWAFHCHIEPHLHMGMGVIFAEAIDRVGKVPKEAVSCGATATALMNGDHL

>TaAAO12
MTRPHTSSSESPLMVHLLLCCTFLLALAAPATTAASVPAPALTPAKQNMTWDVEYILWSPDCQQRVMIGINGKF
PGPNITARAGETLSITVNNKLHTEGLVIHWHGMRQVGTPWADGTASISQCAISPGDSFTYEFVADKPGTYFYHG
HFGMQRAAGLYGWLVVNATAEQDEPYRRDYDGGELRMLLSDWYHDNVYAQAAGLEQKYDHFQWVGEPQT
ILINGRGQYDCMLGAVTRFHRGIDRRARTCVRGKEAKLCGDEERCLRRSECGPYCPQSQCAPVVFDVEPGRTYR
LRIASTTSLSALNVQVQGHDLTVVEADGNPVEPFTVPDIDIYSGESYSVLLTTNHTPTFYRSGSFWISVGVRGRPP
KTLPATAILRYTNSRFPWPGSPPPATPAWYDLQRSKDFTHRINARRNAAEAPPPPRTEQVNRTIVMLNTQTLVG
GHMKWAVNNVSLTLPATPYLGAYFYGVQGSAFDASGEAPNGFPGGYDIDLPPANNSYETTLSDRVYELPHGAV
VDVVLQNADMRRDNDSETHPWHLHGHDFWVLGYGEGRYTSGRERLNTEDPPLRNTVVVFPHGWTAIRFVA
DNVGAWAFHCHIEPHLHMGMGAVFVEGAHKIRELDVPREAMMCGVIRTTAAALTPAKPGSSAPAPSAHR

>StAAO1
MSSSRLLLLLLVSFMLVGHSLAKTRHFKWEVGYIHWSTDGEESVVMGINGAFPGPTIRGRAGDIIVVELTNKLHT
EGVVIHWHGIRQFGTPWADGTAAISQCAINAGETFVYRFKVDKAGTYFYHGHYGMQRSAGLYGSLIVDVAQG
EREPFHYDGEFNLLLSDWWHKGSHEQEVDLSSNPLRWIGEPQTLLINGRGQYNCSMAAQFSNPRLPQCKLRG
GEQYAPQILRVRPNKTYRLRLASTTALASLNLAIGGHKMVVVEADGNYVQPFSVQDIDIYSGESYSILFTTDQDP
SKNYWISTSVRGREPKTPQALTLLNYLPNSASKVPTLPPPIAPLWNDYNHSKSFSNKILALMGSPKPPIKNNRRIIL
LNTQNKIDGYTKWAINNVSLVLPTTPYLGSIRYGINNAFDTRPPPDNFPIDYDVMKQAPNSNATYGNGVYMLK
MNNTIDIILQNANALGKGASEIHPWHLHGHDFWVLGYGEGKFSEKHVKKFNLKNPPLRNTVVIYPYGWTALRF
VTDNPGVWAFHCHIEPHLHMGMGVILAEGVHLVKNIPREALACGLTGKMLMTNKHN*

>StAAO2

MSSLLGIFLYLFLLLSSISAKNREYKWRVEYMHWCPDGVDGVVISINGQFPGPTIRAIVGDTIFVHLTNNLPTEGL
VIHWHGISQIGTPWADGAALISQCPINPGETFLYKFKVDKAGTYFYHGHYGMQRSAGLYGSLIVEDEKEAFHYD
EEFNLLLSDWWHKSSHDQQIDLSSKPFRWIGEPHTLLMNGRGQFNCSLAAQFSKLPITQCKLRGDEQYAPQIF
KVHPSKTYRLRVASTTALASLNLAIEGHKMMVVEADGNYIQPFFVENMDIYSGESYSILFKTDQDPSNNYWISIS
VRGREPKTPQGLTILNYIPNFASKIPNSPPPLAPLWNDYNYSKAFSNKIFGLMGLSPKPPTRQNRRILLLNTQNRI
EGYIRWSINNISLVFPTTPYLGSIKHGINNAFDTKSPPNTFSKNYNIMKPPPNPNSTYGNRIYMLKFNTTIDIILQN
ANALGENVSEIHPWHLHGHNFWVLGYGDGKFDHKKDVEKFNLKNPPLRNTVVIFPYGWTAIRFVANNPGVW
AFHCHIEPHLHLGMGVVFAEGVHLVKNMPNEALNCGLTRKMFVKDKH*

>StAAO3
MVEHNFHQQIHSFVKLVIFLCLLFLSANISVEARIRHYEWEVKYEYKSPDCFKKLSISINGTTPGPTIVAQQGDTIV
VEVKNSLLTENLAIHWHGIRQIGTPWADGTEGVTQCPIVPGDTFVYKFVVDRAGTYLYHAHYGMQRQAGLQG
MIRVSLPDGVLEPFSYDHDKSILLTDWYHKSTYEQATGLASLPFSWVGEPQSILIHGRGRFNCSIPSIDPTLCNAT
NPQCTPYSMTVVSGKTYRLRIGSLTALSALSFEIEGHNMTVVEADGHYVEPFVVKNLFIYSGETYSVLIKADQDPT
RNYWASTKIVSRNSTTPNGLGIFNYYPNHPRRYPPSVPPSGPRWDDVAPRMAQSVAIKSHKDFIHAPPLTSDRV
IVMLNTQNRVNGYVRWSVNNVSFNMPHTPYLIALKHNLLHTFEQTPPPDNYDHKNYDIFNIAPNVNATTSNSI
YRLKFNTTVDIILQNANTMNLNNSETHPWHLHGHDFWVMGYGNGKFNQSIDPKNYNFVNPIMKNTIPVHPY
GWTALRFRADNPGVWAFHCHIESHFFMGMGVVFEEGIEKVGKLPTSIMGCGESKRFLRP*

>StAAO4
MSWICPADQLTPIRAYLDQKLDGEGMSEPNFKRRLKREFVIMNGRLNMNTNHQIVSRNFLLVSMVKLQDLLLL
LNKVTLLLLKYKNSLLTENLAIHWHGIRQIGTPWADGTEGVTQCPIVPGDTFVYKFVVDRAGTYLYHAHYGMQR
QAGLQGMIRVSLPDGVLEPFSYDHDKSILLTDWYHKSTYEQATGLASLPFSWVGEPQSILIHGRGRFNCSIPSIDP
TLCNATNPQCTPYSMTVVSGKTYRLRIGSLTALSALSFEIEGHNMTVVEADGHYVEPFVVKNLFIYSGETYSVLIK
ADQDPTRNYWASTKVVSRNNTTPNGLGIFNYYPNHPRRYPPTVPPPGPRWDDVTPRMAQSVAIKSHKDFIHA
PPLTSDRVIVMLNTQNRVNGYVRWSVNNVSFNMPHTPYLIALKHNLLHTFEQTSPPDNYDHKNYDIFNIAPNV
NATTSNSIYRLKFNTTVDIILQNANTMNLNNSETHPWHLHGHDFWVMGYGNGKFNQSIDPKNYNFVNPIMK
NTIPVHPYGWTALRFRADNPGVWAF HCHIESHFFMGMGVVFEEGIEKVGKLPTSIMGCGESKRFLRP*

>StAAO5
MGNVALFHLICGIFIFWSVSVVKAEDTYKYFTWTATYGTLSPLGVPQQVILINGQFPGPRLDLVTNDNVILNLINK
LDEPLLLTWNGIKQRKNSWQDGVLGTNCPIPINSNYTYKFQTKDQIGSYTYFPSTQLHRAVGGFGALNVYARSVI
SVPYAKPAGDFSLLIGDWYKSSHKVLRQILDSGRSLPYPNGLLINGQKQSTFSGDQGKTYMFRISNVGLKNSINF
RIQGHKMRVVEVEGSHVLQNFYDSLDVHVGQSMSVLVTLDQPPKDYYIVASTRFSRINFTATSVLHYTNSQTPV
SGPMPPAPAGQMHWSMLQARTFRWNLTSNAARPNPQGSFHYGKITISRTFVMANSAPLINGKLRYAVNSVS
YVNPDTPLKLADHFNIPGVFNLNSIQAYPSGGSPYLGTAVFPTSHHDFIEIVFQNDEATMQSWHLDGYDFWVV
GFGSDKWTQASRNKYNLVDALTRHTTQVYPKSWTAILVSLDNQGMWNLRSAMWDRQYLGQQVYLRVYDPT
PSLANEYDIPTNALLCGKAAGRHT*

>StAAO6
MGKLALLHLLCGILIFWAVSVIKAEDPYKYFTWTATYGMASLLGTPQQVILINGQFPGPRLDLVTNDNIVLNLINK
LDEPLLLTWNGIKQRKNSWQDGVLGTNCPIPPNRNYIYKFQPKDQIGSYTYFPSTQLHRAAGGFGVLNVYARSV
IPVPYAKPAGDFSLLIGDWYKTSHKALRQILDSGKSLPFPDGLLINGQKQSTFTGDQGKTYMFRISNVGLKTTINF
RIQGHKMKLVEVEGSHVIQNLYDSFDVHVGQSLSILVTLDQSPKDYYIVASTRFTTTVLTSTSVLHYTNSQTPV

SGPVPAGPVNQFQWSLMQARTIRWNLTSNAARPNPQGSFHYGKITPSRTILLANSAPLINGKQRYAVNGISYIH
PDTPLKLADHFNIPGVFTLDSIRSAPTADSPHLATAVLPSSLHEFLEIVFQNNEDTMQSWHLDGYDFWVVGFGS
GTWTQASRKGYNLVDALTRHTTQVYPKSWTAIFVSLDNQGMWNLRSAMWDRQYLGQQAYLRVYNPTSSLA
NEYDIPTNALLCGKADALRHP*

>StAAO7
MHWCPDGVDGVVISINGQFPGPTIRAIVGDTIFVHLTNNLPTEGLVIHWHGISQIGTPWADGAALISQCPINPG
ETFLYKFKVDKTLLMNGRGQFNCSLAAQFSKLPITQCKLRGDEQYAPQIFKVHPSKTYRLRVASTTALASLNLAIE
GHKMMVVEADGNYIQPFFVENMDIYSGESYSILFKTDQDPSNNYWISISVRGREPKTPQGLTILNYIPNFASKIP
NSPPPLAPLWNDYNYSKAFSNKIFGLMGLSPKPPTRQNRRILLLNTQNRIEGYIRWSINNISLVFPTTPYLGSIKH
GINNAFDTKSPPNTFSKNYNIMKPPPNPNSTYGNRIYMLKFNTTIDIILQNANALGENVSEIHPWHLHGHNFW
VLGYGDGKFDHKKDVEKFNLKNPPLRNTVVIFPYGWTAIRFVANNPGVWAFHCHIEPHLHLGMGVVFAEGVH
LVKNMPNEALNCGLTRKMFVKDKH*

>StAAO8
MGNVALFHLICGIFIFWSVSVVKAEDTYKYFTWTATYGTLSPLGVPQQVILINGQFPGPRLDLVTNDNVILNLINK
LDEPLLLTWNGIKQRKNSWQDGVLGTNCPIPINSNYTYKFQTKDQIGSYTYFPSTQLHRAVGGFGALNVYARSVI
SVPYAKPAGDFSLLIGDWYKSSHKVLRQILDSGRSLPYPNGLLINGQKQSTFSGDQGKTYMFRISNVGLKNSINF
RIQGHKMRVVEVEGSHVLQNFYDSLDVHVGQSMSVLVTLDQPPKDYYIVASTRFSRINFTATSVLHYTNSQTPV
SGPMPPAPAGQMHWSMLQARTFRWNLTSNAARPNPQGSFHYGKITISRTFVMANSAPLINGKLRYAVNSVS
YVNPDTPLKLADHFNIPGVFNLNSIQAYPSGGSPYLGTAVFPTSHHDFIEIVFQNDEATMQSWHLDGYDFWVV
GYVITLQNISISPCYITVEIRSNPHLLLLICSFGSDKWTQASRNKYNLVDALTRHTTQVYPKSWTAILVSLDNQGM
WNLRSAMWDRQYLGQQVYLRVYDPTPSLANEYDIPTNALLCGKAAGRHT*

>StAAO9
MGKLALLHLLCGILIFWAVSVIKAEDPYKYFTWTATYGMASLLGTPQQVILINGQFPGPRLDLVTNDNIVLNLINK
LDEPLLLTWNGIKQRKNSWQDGVLGTNCPIPPNRNYIYKFQPKDQIGSYTYFPSTQLHRAAGGFGVLNVYARSV
IPVPYAKPAGDFSLLIGDWYKTSHKALRQILDSGKSLPFPDGLLINGQKQSTFTGDQGKTYMFRISNVGLKTTINF
RIQGHKMKLVEVEGSHVIQNLYDSFDVHVGQSLSILVTLDQSPKDYYIVASTRFTTTVLTSTSVLHYTNSQTPVSG
PVPAGPVNQFQWSLMQARTISNNKGSNYHENTSERWNLTSNAARPNPQGSFHYGKITPSRTILLANSAPLING
KQRYAVNGISYIHPDTPLKLADHFNIPGVFTLDSIRSAPTADSPHLATAVLPSSLHEFLEIVFQNNEDTMQSWHLD
GYDFWVVGFGSGTWTQASRKGYNLVDALTRHTTQVYPKSWTAIFVSLDNQGMWNLRSAMWDRQYLGQQA
YLRVYNPTSSLANEYDIPTN ALLCGKADALRHP
